# Red rot resistant transgenic sugarcane developed through expression of *β-1*,*3-glucanase* gene

**DOI:** 10.1371/journal.pone.0179723

**Published:** 2017-06-28

**Authors:** Shivani Nayyar, Bipen Kumar Sharma, Ajinder Kaur, Anu Kalia, Gulzar Singh Sanghera, Karanjit Singh Thind, Inderjit Singh Yadav, Jagdeep Singh Sandhu

**Affiliations:** 1 School of Agricultural Biotechnology, Punjab Agricultural University, Ludhiana, India; 2 Punjab Agricultural University, Regional Research Station, Kapurthala, India; 3 Electron Microscopy and Nanoscience Laboratory, Punjab Agricultural University, Ludhiana, India; 4 Department of Plant Breeding and Genetics, Punjab Agricultural University, Ludhiana, India; Bhabha Atomic Research Centre, INDIA

## Abstract

Sugarcane (*Saccharum* spp.) is a commercially important crop, vulnerable to fungal disease red rot caused by *Colletotrichum falcatum* Went. The pathogen attacks sucrose accumulating parenchyma cells of cane stalk leading to severe losses in cane yield and sugar recovery. We report development of red rot resistant transgenic sugarcane through expression of *β-1*,*3-glucanase* gene from *Trichoderma* spp. The transgene integration and its expression were confirmed by quantitative reverse transcription-PCR in first clonal generation raised from T_0_ plants revealing up to 4.4-fold higher expression, in comparison to non-transgenic sugarcane. Bioassay of transgenic plants with two virulent *C*. *falcatum* pathotypes, Cf 08 and Cf 09 causing red rot disease demonstrated that some plants were resistant to Cf 08 and moderately resistant to Cf 09. The electron micrographs of sucrose storing stalk parenchyma cells from these plants displayed characteristic sucrose-filled cells inhibiting Cf 08 hyphae and lysis of Cf 09 hyphae; in contrast, the cells of susceptible plants were sucrose depleted and prone to both the pathotypes. The transgene expression was up-regulated (up to 2.0-fold in leaves and 5.0-fold in roots) after infection, as compared to before infection in resistant plants. The transgene was successfully transmitted to second clonal generation raised from resistant transgenic plants. β-1,3-glucanase protein structural model revealed that active sites Glutamate 628 and Aspartate 569 of the catalytic domain acted as proton donor and nucleophile having role in cleaving β-1,3-glycosidic bonds and pathogen hyphal lysis.

## Introduction

Sugarcane (*Saccharum* spp.) is a commercially important crop of tropical and sub-tropical regions cultivated primarily for production of sucrose, ethanol, biofuel and fibre-related commodities [1, 2]. The crop is vulnerable to several diseases and maximum devastation occurs due to red rot caused by fungus *Colletotrichum falcatum* Went [3]. The pathogen attacks sucrose accumulating parenchyma cells [4] of economically important cane stalk [5] leading to severe losses in sugar recovery (25–75%), extraction (7.1–32.5%), polarity (7.4–38.7%), purity co-efficient (0.5–8.3%), and commercial cane sugar (7.8–39%) [6, 7].

*C*. *falcatum* spreads through infected cane stalk setts, diseased debris, resting spores in soil, and the disease can be avoided in this vegetatively propagated crop through the use of healthy planting material [8]. The disease management by chemical treatment is reported to be difficult [9], whereas the use of biological agents, such as *Trichoderma* spp. has been demonstrated to control red rot in sugarcane [10, 11]. *Trichoderma* secretes various cell wall-degrading enzymes, among these β-1,3-glucanase targets β-glucan chain in *Colletotrichum* cell wall at β-1,3-glucosyl linkages [12, 13] resulting in swelling, distortion, lysis of pathogen hypha [14, 15], thus restricting their growth and activity [16]. The antifungal activity of *Trichoderma* spp. β-1,3-glucanase has been shown to inhibit the growth of a number of fungal pathogens and has been directly associated with disease resistance [17]. The cloning and characterization of *β-1*,*3-glucanase* genes, such as *bgn*13.1 [18] and *gluc*78 [19] from *Trichoderma* spp. demonstrated inhibitory activity of the genes against phytopathogenic fungi. Further, their over-expression in transgenic plants resulted in imparting fungal resistance e.g. *bgn*13.1 expression in *Brassica napus* provided enhanced glucanase activity against *Sclerotinia sclerotiorum* [20], and displayed tolerance against *C*. *acutatum* and *Rosellinia necatrix* in strawberry [21]; likewise, *gluc*78 expression in rice provided resistance against *Magnaporthe grisea* [22], and *Sclerospora graminicola* in pearl millet [23]. However, there is no information in literature about expression of *β-1*,*3-glucanase* for resistance against fungal pathogens in sugarcane.

This study was undertaken to develop transgenic sugarcane using *β-1*,*3-glucanase* gene for providing resistance against red rot. The antifungal gene was isolated from local *Trichoderma* spp. that exhibited significant similarity with other genes encoding β-1,3-glucanase in the GenBank database. The first clonal generation of T_0_ plants was analyzed for integration, expression of the transgene and transgenic plants were bioassayed with two virulent *C*. *falcatum* pathotypes for red rot incidence. The sucrose storing stalk parenchyma cells of these plants were electron micrographed to study the incidence of fungal hyphae.

## Materials and methods

### Preparation of plant material

The stalks were collected from 10 month-old healthy plants of sugarcane cultivar CoJ 83 grown in the field area of School of Agricultural Biotechnology (SOAB), Punjab Agricultural University (PAU), Ludhiana, Punjab, India during the main cropping season (March-December, 2013). The plant material for *Agrobacterium* transformation was prepared by cutting the stalks into billets (2–3 cm long), each carrying one axillary bud. The billets were treated with 1% bavistin (w/v) for 10 min with gentle shaking at 100 rpm on an orbital shaker (New Brunswick Scientific, USA) followed by two washes with distilled water; surface sterilized in 0.1% mercuric chloride (w/v) for 10 min and finally washed thrice with distilled water. The billet disinfection was carried out aseptically in a laminar air flow cabinet (Klenzoids, India).

### Gene construct and *Agrobacterium* strain

**Fig 1:**
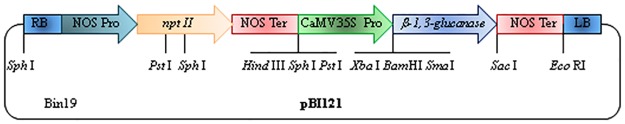
Map of gene construct. The 1764 bp *β-1*,*3-glucanase* catalytic domain driven by CaMV 35S promoter and NOS terminator in pBI121.

The gene construct containing 1764 bp *β-1*,*3-glucanase* catalytic domain (GenBank accession no. KJ603460) driven by CaMV 35S promoter and NOS terminator was assembled in the binary vector pBI121 (GenBank accession no. AF485783) [Fig 1]. The construct was mobilized into *Agrobacterium tumefaciens* strain LBA4404 through tri-parental mating. The transformed LBA4404 strain carrying the gene construct was streaked on YEMKS medium [Yeast extract (0.4 g/l), mannitol (10 g/l), NaCl (0.1 g/l), MgSO_4_.7H_2_O (0.2 g/l), K_2_HPO_4_.3H_2_O (0.5 g/l), agar (16 g/l) supplemented with kanamycin (50 mg/l) and streptomycin (50 mg/l)] and incubated at 28°C for 36 h. A single colony of transformed LBA4404 was lifted, suspended in liquid YEMKS medium and grown at 28°C by shaking (100 rpm) on an orbital shaker. The bacterial culture was quantified spectrophotometrically (Hitachi, Japan) at 600 nm to obtain an optical density of 1.0–1.2 cfu/ml, pelleted by centrifugation (Remi, India) at 5000 rpm and suspended in liquid MS medium (30 ml; pH 5.8) supplemented with acetosyringone (9.8 mg/l). This aseptically prepared *Agrobacterium* suspension was used for in planta genetic transformation of sugarcane.

### *In planta* transformation

Axillary bud on each sterilized billet was pricked (1 mm in depth) five times randomly using a sterile needle (Dispovan, India). Six billets carrying pricked axillary buds (PABs) were placed in a sterile beaker (1000 ml, Borosil, India) and immersed in diluted *Agrobacterium* suspension (600 ml i.e. 30 ml suspension in 570 ml liquid MS medium) for 5 h at 28°C. The agro-infected PABs were blotted dry using sterile filter paper (Whatman, USA) and co-cultivated on basal MS medium in jam jars at 28°C for 72 h. The co-cultivated PABs were washed in sterile water containing cefotaxime (500 mg/l) for 20 min, blotted dry and sown (one billet per bag) in polythene bags (12 cm x 20 cm) containing potting mixture (soil and farm yard manure in 3:1 ratio) with buds facing upwards. The PABs in polythene bags were incubated in transgenic glass house, watered twice a day during summers and once a day during winters for plant development. The three month-old putative T_0_ plants were transferred to earthen pots (22.5 cm diameter) containing potting mixture and grown to maturity. Subsequently, first clonal generation (CG_1_) and second clonal generation (CG_2_) of T_0_ plants were raised in plant to row fashion through billet planting in earthen pots as the crop does not set seed in this geographical region.

### Polymerase chain reaction

The 45 day-old putative plants were screened for the presence of *β-1*,*3-glucanase* transgene through PCR. The genomic DNA was extracted from newly emerging leaves of putative and non-transgenic (NT) plants according to CTAB method described by Murray and Thompson [24] and used as a template in PCR. The transgene specific primers 5'-ATGTTGAAGCTCACGGCGCTCGTTG-3' and 5'-ACTCGATTGCAGGGAAAGGCGGA-3' were designed to obtain 1764 bp amplicon. The PCR mixture (20 μl) contained 100 ng genomic DNA (2 μl), 10 μM of each primer (1 μl), 1 mM dNTPs (4 μl), 25 mM MgCl_2_ (1.5 μl), 5X Green GoTaq^®^ flexi buffer (4 μl), 5 units GoTaq^®^ DNA polymerase (1 μl) [Promega, USA] and nuclease free water (5.5 μl). The reactions were performed in a thermal cycler (Eppendorf Master Cycler, Germany) programmed for an initial denaturation at 95°C for 5 min, followed by 30 cycles of denaturation at 95°C for 1 min, annealing at 60°C for 2 min, extension at 72°C for 2 min, and concluded by a final extension at 72°C for 10 min. The NT genomic DNA was used as negative control. The amplified products were electrophoresed on 1% (w/v) agarose gel at 80 V for 2 h, stained with ethidium bromide, visualized under UV light and photographed using gel documentation system (Avegene, USA).

### RNA isolation and cDNA synthesis

The total RNA was isolated from newly emerging tender leaves and roots (50 mg fresh weight) of putative PCR positive and NT plants in TRI Reagent^R^ (Sigma-Aldrich, USA) using liquid nitrogen following the method given by Chomczynski and Sacchi [25]. The RNA samples were treated with DNase I of RNA easy kit (Qiagen, Germany) and quantified spectrophotometrically at 260/280 nm. The verification of RNA integrity was carried out on 1.2% (w/v) denaturing agarose gel prepared in 1X MOPS buffer (200 mM MOPS, 80 mM sodium acetate and 10 mM EDTA). The electrophoresed samples were visualized as described earlier. RNA concentration was adjusted to 1 μg/μl, and cDNA strand was synthesised using GoScript^™^ Reverse Transcription System (Promega) according to manufacturer’s instructions.

### Semi-quantitative RT-PCR

The cDNA of putative PCR positive plants was analysed for the presence of 534 bp *26S rRNA* gene (GenBank accession no. AY283368) [26] using gene specific primers 5'-CACAATGATAGGAGGAGCCGAC-3' and 5'-CAAGGGAACGGGCTTGGCAGAATC-3'. The RT-PCR mixture (20 μl) comprised of 30 ng cDNA (3 μl), 10 μM of each primer (1 μl), 1 mM dNTPs (4 μl), 25 mM MgCl_2_ (1.5 μl), 5X Green GoTaq^®^ flexi buffer (4 μl), 5 units GoTaq^®^ DNA polymerase (1 μl) and nuclease free water (4.5 μl). The thermal cycler was programmed at an initial denaturation of 95°C for 5 min, followed by 30 cycles at 95°C for 1 min, annealing at 60°C for 2 min, extension at 72°C for 2 min and a final extension at 72°C for 5 min. The amplicons were electrophoresed and visualized as described earlier for cDNA integrity confirmation. Subsequently, RT-PCR was performed using *β-1*,*3-glucanase* gene specific primers to study transcription of the transgene.

### Real time quantitative RT-PCR

The real time quantitative RT-PCR was carried out on leaves of RT-PCR positive and NT plants for identifying transgenic plants with relatively high *β-1*,*3-glucanase* expression using transgene specific qRT-PCR primers 5'-CTCTCCAGAATGCTATCACCAC-3' and 5'-GGTATATGTTCCCGGAGGAATG-3', and internal control *β-tubulin* gene (GenBank accession no. CA222437) [27] specific primers 5'-CCAAGTTCTGGGAGGTGATCTG-3' and 5'-TTGTAGTAGACGTTGATGCGCTC-3'. The qRT-PCR mixture (20 μl) contained 2X PCR SYBR Green supermix (10 μl), cDNA template (2 μl), 10 μM each primer primer (1 μl) and sterile distilled water (6 μl). The qRT-PCR was carried out on 96 Real-Time PCR System (Roche LightCycler^®^, Germany) programmed at 95°C for 3 min, followed by 40 cycles at 95°C for 10 s, 60°C for 30 s, 72°C for 30 s and 81 cycles at 55–95°C for 30 s to analyze the expression of *β-tubulin* and *β-1*,*3-glucanase* genes. The serial dilutions (10-fold) of cDNAs were used to generate standard curves. The assays were performed in triplicates and data were analyzed using CFX manager 3.0 software. Amplification efficiencies of both genes in qRT-PCR were determined by plotting cDNA dilution against C_T_ value and are shown in Figs A and B in S1 File. The amplification chart for *β-tubulin*, *β-1*,*3-glucanase* genes is shown in Fig C in S1 File and melt curve peaks for the two genes are shown in Fig D of S1 File. *β-1*,*3-glucanase* expression in RT-PCR positive plants was normalized against *β-tubulin* gene. The accuracy and reproducibility of the realtime assay was determined from low variation in C_T_ values across replicates (Table A of S1 File). The fold change in transgene expression was determined in comparison to NT plant using 2^-ΔΔC^_T_ method [28].

### Bioassay for red rot

Bioassay for red rot incidence was carried out with Cf 08 and Cf 09 *C*. *falcatum* pathotypes. The two pathotypes were collected from red rot affected sugarcane cultivars CoJ 64 and CoS 767, respectively by Division of Crop Protection, Indian Institute of Sugarcane Research, Lucknow, Uttar Pradesh, India during 2014–15. The cultures were raised individually and aseptically on potato dextrose agar (PDA) petriplates (90 mm; Tarsons, India) under continuous light at 28°C for 10 days [29]. Sterile distilled water (10 ml) was added to each culture petriplate and colonies on PDA surface were scraped with a scalpel. The conidial suspensions were filtered through sterile cheese cloth and final concentration was adjusted to 70,000 conidia/ml with sterile distilled water. The primary stalks of four month-old transgenic and NT plants were plug inoculated [30] by dropping fungal suspension (1 ml) of Cf 08 with a syringe into a bore hole made at the 3^rd^ stalk internode from bottom, the hole was re-plugged with removed stalk tissue and tightly sealed with parafilm (Tarsons). The plants were kept under high humidity (>80%) in the transgenic glasshouse for disease development, monitored at weekly intervals and evaluated for disease symptoms by split opening the inoculated stalks longitudinally after an incubation period of eight weeks. The disease severity was scored on basis of condition on top, red rot lesions along the stalk length, width of the lesions, nodal transgression and white spots present in the inoculated stalks. The cumulative value for all symptoms was recorded on 0–9 scale [31]. The stalks were cut near the base after recording disease data; in the subsequent year, ratoon transgenic and NT plants were plug inoculated with Cf 09 and analyzed for red rot incidence. The leaves, roots of these plants were analyzed for relative *β-1*,*3-glucanase* expression before inoculation with Cf 09 and six weeks after inoculation using transgene specific and *β-tubulin* gene specific primers following qRT-PCR conditions as described earlier. The amplification chart, melt curve peaks before inoculation in leaves are shown in Figs E and F of S1 File, respectively; in roots are shown in Figs G and H of S1 File, respectively. The amplification chart after inoculation in leaves and roots is shown in Fig I of S1 File, and melt curve peaks in Fig J of S1 File. The C_T_ values across replicates in leaf samples before and after inoculation (Tables B and C of S1 File, respectively) and root samples before and after inoculation (Tables D and E of S1 File, respectively) were calculated to verify the precision and reproducibility of the assay. The fold change in transgene expression was obtained using 2^-ΔΔC^_T_ method.

### Scanning electron micrography

The stalks of inoculated and non-inoculated plants were sectioned transversely above (3 to 4 cm) the bore hole for obtaining 0.5 to 1 cm thick discs. The discs were sub-sectioned, pre-fixed in modified Karnovsky’s reagent (2% paraformaldehyde + 2.5% glutaraldehyde) for 24 h at 4°C [32], washed in phosphate buffer and post-fixed in osmium tetraoxide for 1 h at 4°C. Subsequently, the sectioned tissue was dehydrated by treatment with graded ascending acetone series (from 30 to 100%), dried and stubbed using double-sided carbon tape. The sections were made conductive by sputter coating with 10–20 nm thick gold layer using ion sputter coater (Hitachi E-1010, Japan), followed by micrography under scanning electron microscope (Hitachi S-3400N, Japan) @ 15 KV acceleration voltage in secondary electron imaging mode.

### Analysis of chimerism

The red rot resistant transgenic plants were billet planted to raise next generation for evaluating chimerism through relative *β-1*,*3-glucanase* expression analysis in leaves using transgene specific and *β-tubulin* gene specific primers following qRT-PCR conditions as described above. The amplification chart, melt curve peaks are shown in Figs K, L, M and N of S1 File. The C_T_ values across replicates are given in Tables F and G of S1 File. The 2^-ΔΔC^_T_ method was used to determine fold change in transgene expression.

### Structural model of active site residues

The protein encoded by *β-1*,*3-glucanase* was modelled using protein databank file 3EQN as template, identified using blast search with Modeller software [33]. The quality of modelled structure was accessed with Ramchandran plot of protein structure build using pdbsum server (http://www.ebi.ac.uk/thornton-srv/databases/pdbsum/Generate.html). The conserved set of active site residues were identified, docked with D-glucose monomer using autodock tool [34] and analyzed through UCSF chimera [35].

## Results

### Polymerase chain reaction analysis for the presence of transgene

The agro-infected pricked axillary buds of sugarcane cultivar CoJ 83 exhibited shoot emergence within 14 days upon transfer to potting mixture leading to T_0_ plant formation in 28 days and maturity in 300 days. These putative T_0_ plants were not characterized and used to raise CG_1_ through billet planting comprising of 127 plants (Table H of S1 File). The genomic DNA of each CG_1_ plant was verified for the presence of *β-1*,*3-glucanase* transgene through PCR using transgene specific primers. An amplicon of 1764 bp corresponding to the transgene was observed in six CG_1_ plants designated as SN1-12, SN1-19, SN6-2, SN6-5, SN10-12 and SN10-13 (Fig O of S1 File; Table H of S1 File). The transgene amplification in only two plants from 20 SN1 plants; two from 13 SN6 plants and two from 26 SN10 plants pointed towards chimerism in CG_1_ (Table H of S1 File). The six putative PCR positive CG_1_ plants were grown to maturity.

### Semi-quantitative RT-PCR analysis for transcription of transgene

**Fig 2:**
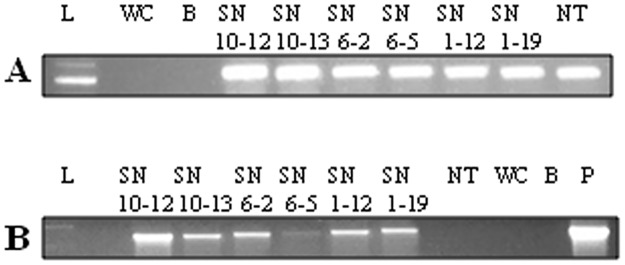
Semi-quantitative RT-PCR analysis on PCR positive CG_1_ plants. **A** cDNA amplification using *26S rRNA* primers. **B** cDNA amplification using *β-1*,*3-glucanase* transgene specific primers. L refers to 1 Kb ladder (Promega, Cat. No. G5711); WC Water Control; B Blank lane; SN10-12, SN10-13, SN6-2, SN6-5, SN1-12 and SN1-19 represent RT-PCR positive plants carrying *β-1*,*3-glucanase* transgene; NT Non-Transgenic, P Plasmid.

The six putative PCR positive CG_1_ plants were promoted for confirming transcription of transgene through semi-quantitative RT-PCR. The total RNA from six CG_1_ plants and NT plant was verified for integrity (Fig P of S1 File). The cDNA analysis with *26S rRNA* gene specific primers resulted in amplification of anticipated 534 bp fragment (Fig 2A), indicating legitimacy of the synthesized cDNA. *26S rRNA* gene was favoured among other housekeeping genes due to abundance (> 80%) and less variation of rRNA under the conditions affecting expression of mRNAs [36]. The reverse transcription of cDNA with *β-1*,*3-glucanase* transgene specific primers established transcription of transgene in six CG_1_ plants (Fig 2B), indicating successful uptake of T-DNA by the plant cells following *Agrobacterium* mediated in planta transformation.

### Real time quantitative RT-PCR analysis for relative transgene expression

**Fig 3:**
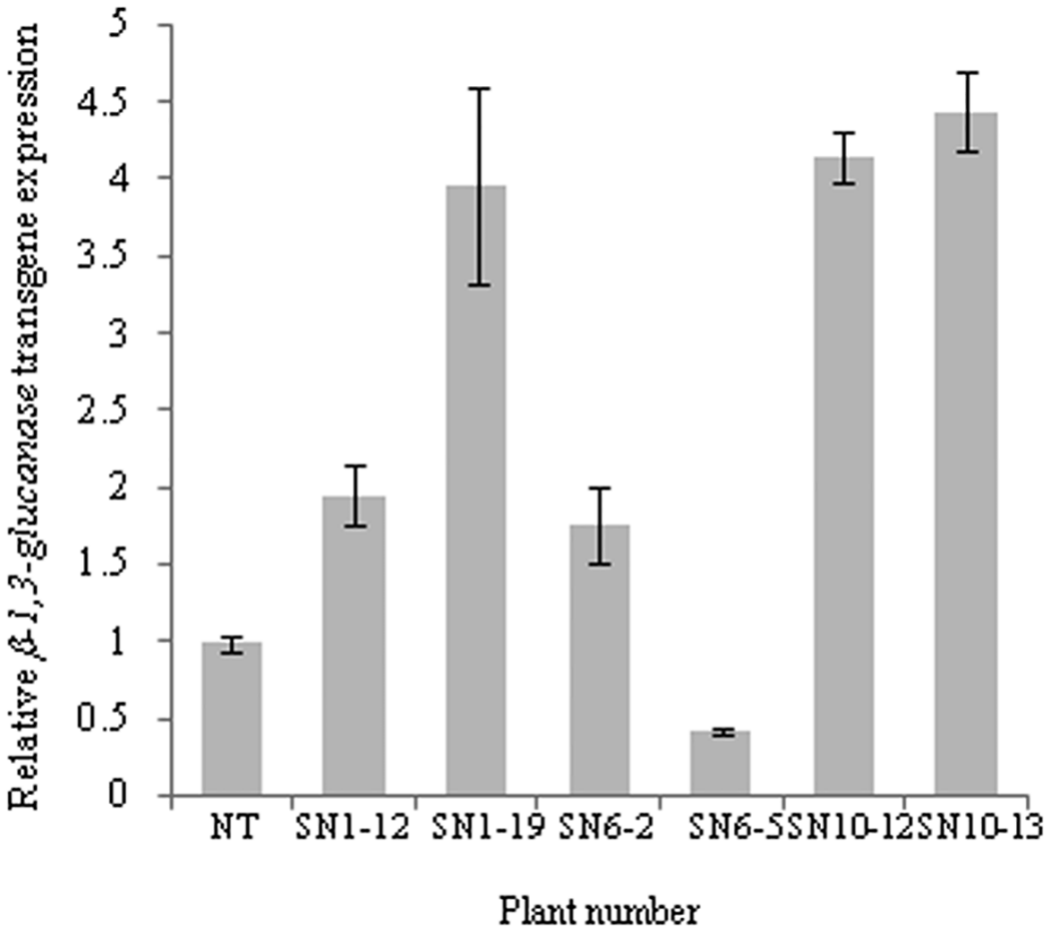
Quantitative RT-PCR analysis. Relative *β-1*,*3-glucanase* expression of RT-PCR positive CG_1_ plants. Bars represent range of 2^- ΔΔC^_T_.

The six RT-PCR positive CG_1_ plants and NT plant were analyzed for relative *β-1*,*3-glucanase* transgene expression through qRT-PCR. The results demonstrated that the plants designated as SN10-13, SN10-12, SN1-19, SN1-12 and SN6-2 had 4.4, 4.1, 3.9, 1.9 and 1.7-fold higher transgene expression, respectively, whereas the expression in SN6-5 plant was down-regulated (0.4-fold) as compared to NT plant (Fig 3; Table I of S1 File). The results confirmed successful integration and expression of *β-1*,*3-glucanase* transgene in the genomes of six CG_1_ transgenic plants.

### Bioassay for red rot incidence

**Fig 4:**
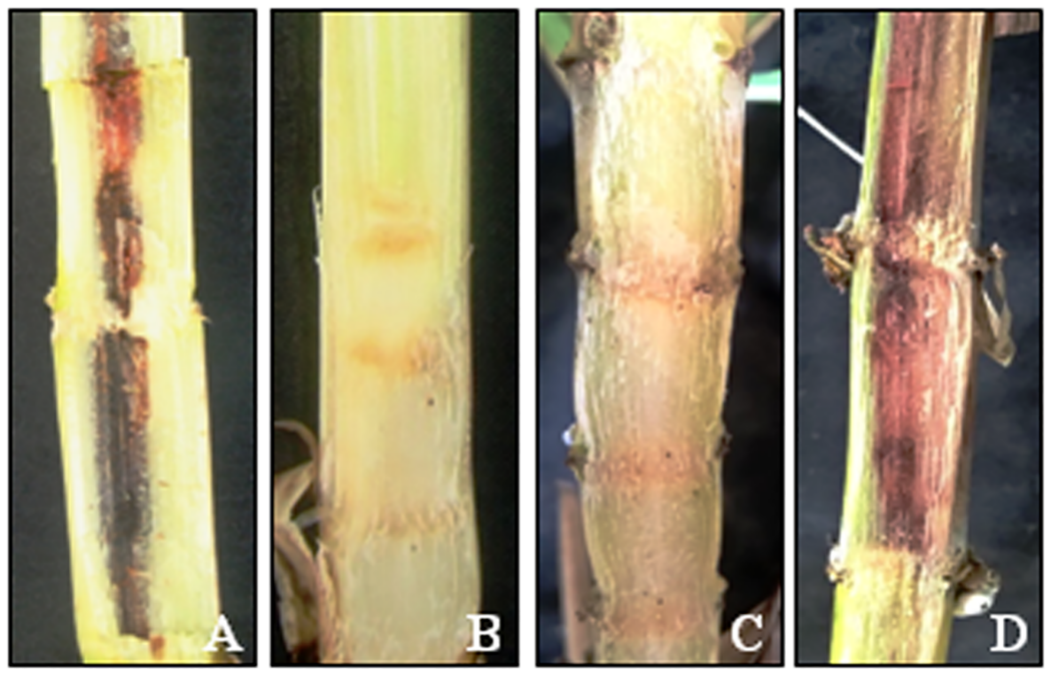
Bioassay for red rot incidence. **A** NT plant inoculated with Cf 08 showing susceptibility. **B** SN10-12 inoculated with Cf 08 revealing resistance. **C** SN10-12 ratoon inoculated with Cf 09 displaying moderate resistance. **D** NT plant inoculated with Cf 09 demonstrating high susceptibility.

The six CG_1_ transgenic and NT plants were evaluated with Cf 08 and Cf 09 pathotypes for red rot incidence. The NT plant inoculated with Cf 08 showed susceptibility symptoms after six weeks revealing presence of sparse white spots in the stalk, red rot lesion transgression across more than two stalk nodes, lesion spread to half the stalk width (Fig 4A) and dry leaves on the top; whereas, in CG_1_ plants designated as SN1-19, SN10-12 and SN10-13, white spots were absent with no lesion transgression (Fig 4B), lesion spread in the inoculated stalk was 25% and leaves on top were green after eight weeks, pointing towards resistance against the pathotype (Table 1). The SN1-12 and SN6-2 plants were moderately resistant to Cf 08 showing transgression of red rot lesion up to two stalk nodes above the site of inoculation, lesion spread to about 25% of the stalk width, green leaves on the top and absence of white spots; SN6-5 plant was susceptible to the pathotype. The ratoon CG_1_ transgenic and NT plants inoculated with Cf 09 revealed SN10-12 and SN10-13 to be moderately resistant (Fig 4C), SN1-12 and SN1-19 to be moderately susceptible, and SN6-2, SN6-5, NT to be highly susceptible to red rot (Fig 4D) [Table 1]. The results confirmed variable response of transgenic plants towards both the pathotypes.

**Table 1:** Bioassay on CG_1_ transgenic plants for red rot incidence.

Plant number	Condition on top^1^	Lesion width^2^	White spots^3^	Nodal transgression^4^	Scale (0–9)^5^	Category^6^
****Inoculation with Cf 08****						
****NT****	0	2	1	3	6	Susceptible
****SN1-12****	0	1	0	2	3	Moderately resistant
****SN1-19****	0	1	0	0	1	Resistant
****SN6-2****	0	1	0	2	3	Moderately resistant
****SN6-5****	0	1	0	3	4	Susceptible
****SN10-12****	0	1	0	0	1	Resistant
****SN10-13****	0	1	0	0	1	Resistant
****Inoculation with Cf 09****						
****NT****	1	2	1	3	7	Highly susceptible
****SN1-12****	1	1	0	2	4	Moderately susceptible
****SN1-19****	1	1	0	2	4	Moderately susceptible
****SN6-2****	1	2	1	2	6	Highly susceptible
****SN6-5****	1	2	1	2	6	Highly susceptible
****SN10-12****	0	1	0	2	3	Moderately resistant
****SN10-13****	0	1	0	2	3	Moderately resistant

Note: The superscript numbers refer to red rot rating scale given by Srinivasan and Bhat [31]

### Real time quantitative RT-PCR analysis before and after inoculation for relative transgene expression

A comparison was made between relative *β-1*,*3-glucanase* transgene expression before inoculation and six weeks after inoculation with Cf 09 pathotype in leaves and roots of ratoon CG_1_ transgenic plants through qRT-PCR analysis. The results revealed that the transgene expression was up-regulated after inoculation in SN10-12 and SN10-13 up to 2-fold in leaves and 5-fold in roots (Table 2; Table J of S1 File), suggesting that increased *β-1*,*3-glucanase* expression was related with pathogen infection. No remarkable increase was observed in the transgene expression level after inoculation in SN6-2 and SN6-5. These results indicated that transgene expression in CG_1_ transgenic plants was differentially influenced after pathogen infection.

**Table 2:** Comparison of relative *β-1*,*3-glucanase* expression in leaves and roots of CG_1_ transgenic plants before and after inoculation with Cf 09 pathotype.

Plant number	2^- ΔΔC^_T_			
	In leaves		In roots	
	Before inoculation	After inoculation	Before inoculation	After inoculation
****NT****	1.07 (0.66–1.49)	0.99 (0.95–1.04)	0.99 (0.96–1.03)	0.99 (0.97–1.02)
****SN1-12****	2.01 (1.73–2.29)	2.37 (2.28–2.47)	1.07 (1.03–1.10)	1.27 (1.22–1.32)
****SN1-19****	3.78 (3.68–3.89)	3.91 (3.69–4.13)	1.59 (1.58–1.60)	1.60 (1.56–1.64)
****SN6-2****	1.60 (1.36–1.85)	1.86 (1.58–2.14)	0.93 (0.90–0.96)	1.64 (1.47–1.81)
****SN6-5****	0.39 (0.36–0.42)	0.14 (0.13–0.14)	0.73 (0.73–0.74)	0.71 (0.65–0.77)
****SN10-12****	4.03 (3.94–4.12)	9.42 (9.35–9.49)	1.61 (1.40–1.82)	8.04 (7.73–8.40)
****SN10-13****	4.24 (4.04–4.44)	7.42 (7.01–7.42)	1.82 (1.80–1.85)	5.94 (5.73–6.14)

### Scanning electron micrography of sucrose storing stalk parenchyma cells for incidence of fungal hyphae

**Fig 5:**
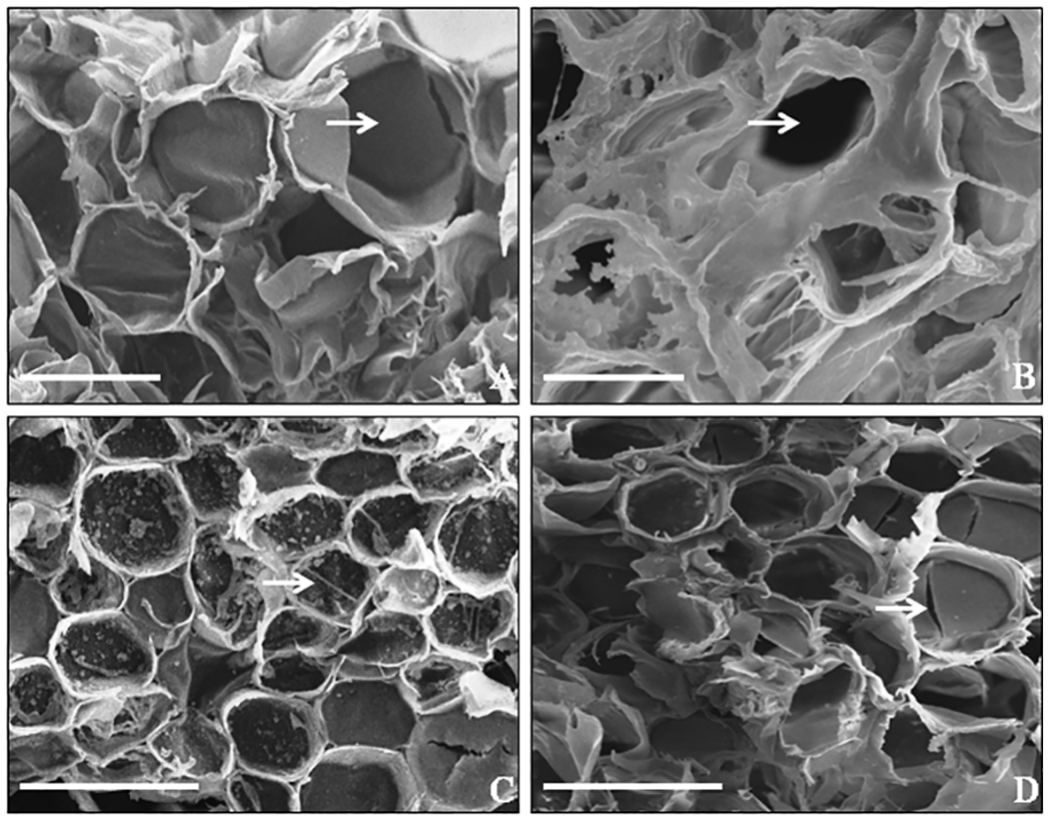
Scanning electron micrographs of stalk sections from CG_1_ transgenic and NT plants following inoculation with *C*. *falcatum*. **A** Sucrose-filled parenchyma cells of non-inoculated NT plant (control). Arrow indicates the characteristic turgid cell. Bar represents 20 μm. **B** Presence of normal Cf 09 fungal hyphae in parenchyma cells of susceptible NT plant. Arrow indicates the sucrose depleted cell. Bar represents 20 μm. **C** Parenchyma cells of transgenic plant moderately resistant to Cf 09. Arrow indicates abnormal fungal hypha and amorphous debris. Bar represents 100 μm. **D** Sucrose-filled parenchyma cells of transgenic plant resistant to Cf 08 showing absence of hyphae. Arrow indicates the turgid cell. Bar represents 100 μm.

The stalk sections of CG_1_ transgenic and NT plants showing resistant, moderately resistant and susceptible reaction to *C*. *falcatum* were analyzed for the incidence of fungal hyphae in sucrose accumulating stalk parenchyma cells. In addition, observations were also recorded on sections of a non-inoculated NT plant. The micrographs of non-inoculated NT plant showed the characteristic sucrose-filled turgid parenchyma cells (Fig 5A). In contrast, the sections of susceptible NT plant inoculated with either Cf 08 or Cf 09 revealed the presence of thin (normal) fungal hyphae in sucrose depleted parenchyma cells (Fig 5B). The sections of transgenic SN10-12 plant (moderately resistant to Cf 09 and resistant to Cf 08) evinced the presence of trailing swollen (abnormal) hyphae of Cf 09 and amorphous debris over the parenchyma cells (Fig 5C), and absence of Cf 08 hyphae (Fig 5D). The parenchyma cells of inoculated SN10-12 were sucrose-filled, intact and comparable to non-inoculated NT plant. Likewise, the sucrose storing cells of SN10-13 also showed restricted growth of Cf 08 hyphae and lysis Cf 09 hyphae. The micrographs confirmed that red rot resistant transgenic plants were successfully inhibiting the incidence of fungal hyphae in sucrose accumulating cells without sucrose loss.

### Analysis of CG_2_ plants for chimerisim

The billets of two red rot resistant CG_1_ transgenic plants (SN10-12 and SN10-13) were used to raise CG_2_. A total of seven CG_2_ plants from SN10-12 and eight from SN10-13 were analysed through PCR, semi-quantitative RT-PCR and quantitative RT-PCR for evaluation of chimeras. The PCR analysis revealed amplification of *β-1*,*3-glucanase* transgene in all CG_2_ plants (Table K of S1 File; Fig Q of S1 File). The integrity of total RNA from fifteen CG_2_ plants was verified (Fig R of S1 File) and semi-quantitative RT-PCR demonstrated transcription of the transgene in these plants (Figs S and T of S1 File). The quantitative RT-PCR analysis revealed that the relative transgene expression was similar in these 15 CG_2_ plants (Table L of S1 File), confirming absence of chimeras and successful transmission of the transgene to second clonal generation.

### Structural model for identification of active site residues in the catalytic domain

**Fig 6:**
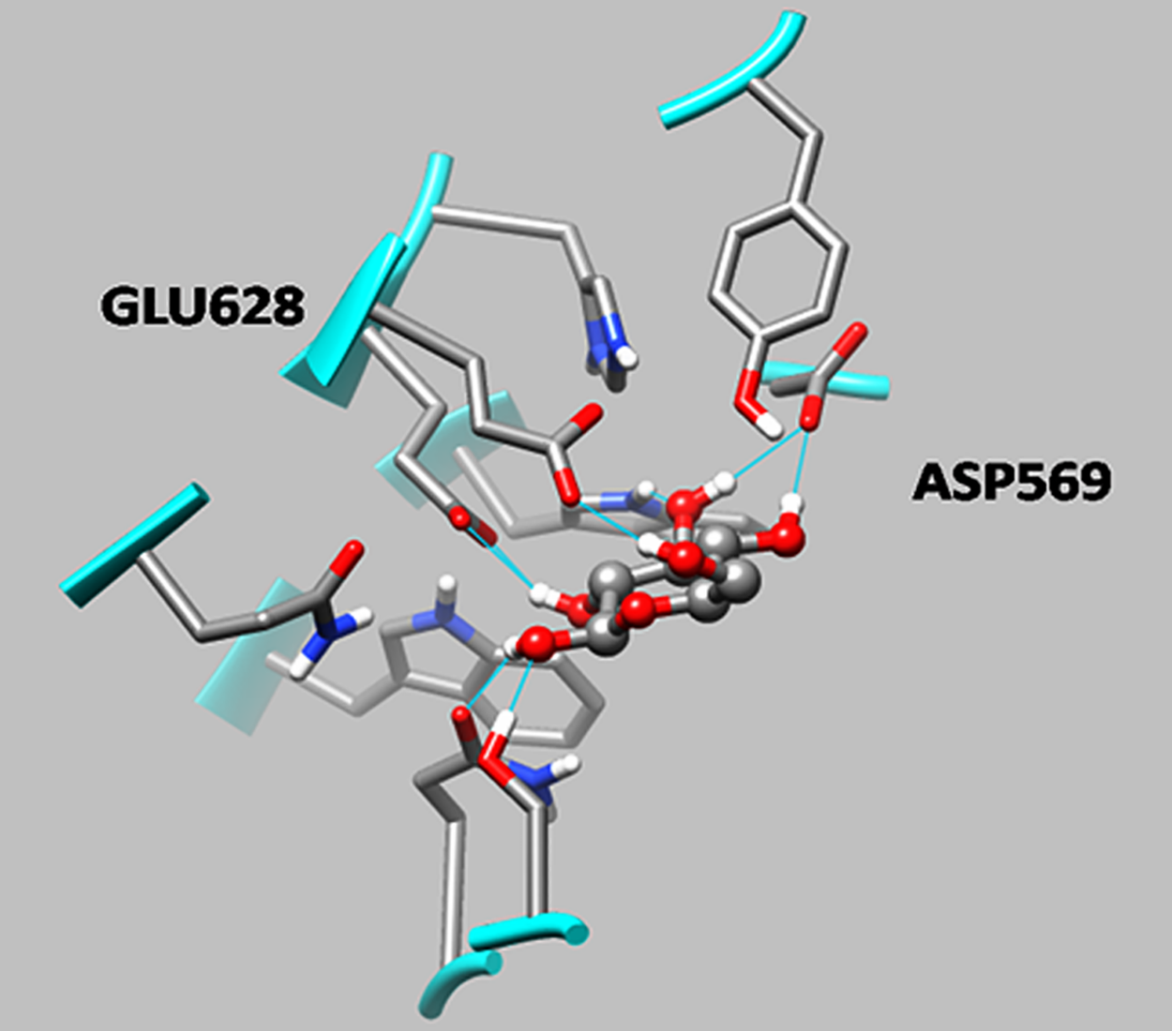
Structural model showing protein-ligand interaction. β-1,3-glucanase protein (GenBank Accession No. AIC32930) docked with D-glucose.

The modelled three dimensional structure of β-1,3-glucanase protein (AIC32930) consisted of 80.9% residues in most favoured region, 17.8% in allowed region and only 1.3% (8 residues) in disallowed region. The comparison of modelled β-1,3-glucanase structure and 3EQN with pdb file 4PEY identified six conserved active site residues in the catalytic domain of the protein (Table M of S1 File). The docking positioned D-glucose monomer between active site residues Glutamate 628 and Aspartate 569 that acted as proton donor and nucleophile (Fig 6).

## Discussion

Transgenic sugarcane expressing *β-1*,*3-glucanase* transgene was developed, that displayed resistance against two *C*. *falcatum* pathotypes causing red rot disease. The sucrose accumulating stalk parenchyma cells of these transgenic plants were intact and sucrose-filled, as trailing of fungal hyphae was either inhibited or hyphae were lysed, the transgene expression was up-regulated after infection; and further, the active site residues involved in cleaving β-1,3-glycosidic bonds of *C*. *falcatum* hyphae were identified.

Red rot resistance was incorporated in CoJ 83, an important commercial sugarcane cultivar of north-west region of the country, with reports of up to 100% red rot incidence [37]. The genetically transformed CoJ 83 was bioassayed with two virulent and genetically diverse *C*. *falcatum* pathotypes (Cf 08 and Cf 09) [38, 39] accountable for epiphytotic outbreaks in the region [29]. Red rot symptoms appeared on most of the transgenic plants; however some displayed resistance, suggesting differential response of the plants towards the pathotypes. Further, the transgenic plants exhibiting resistant reaction against Cf 08 displayed moderate resistance to Cf 09, indicating the latter to be more virulent. Differential host pathogen interaction studies by Viswanathan [40] also confirmed that Cf 09 was more virulent as compared to Cf 08 in the north-west region. The plants carrying red rot resistance had relatively high *β-1*,*3-glucanase* transgene expression that was further up-regulated up to 2.0-fold in leaves and 5.0-fold in roots after *C*. *falcatum* infection as compared to before infection, pointing towards role of the transgene in plant defence mechanism in response to pathogen attack. *β-1*,*3-glucanase* expression has been demonstrated to be directly correlated with controlling disease incidence, such as in transgenic pearl millet expressing *gluc*78 [23] and transgenic canola carrying *bgn*13.1 [20]; and the transgene was induced upon pathogen infection [23]. The integration of *β-1*,*3-glucanase* provided resistance to blast in transgenic rice [22], inhibited stem rot in transgenic conola [20] and improved tolerance to collar rot diseases in transgenic strawberry [21], suggesting efficacy of *β-1*,*3-glucanase* in disease control [17]. The glucanases from *Trichoderma* spp. usually exhibit high antifungal activity [21]; however, increased glucanase activity in transgenic strawberry, rice resulted in reduced plant vigour and yield [21, 22]. In contrast, red rot resistant transgenic sugarcane having high *β-1*,*3-glucanase* expression were phenotypically normal and comparable to NT sugarcane, likewise transgenic canola expressing *bgn13*.*1* had normal growth [20].

Breeding for red rot resistance in sugarcane has been difficult through conventional and genetic mapping approaches due to highly heterozygous polyploid crop genome along with narrow genetic base. Though differentially expressed sequence tags have been identified in response to *C*. *falcatum* infection [41, 42], these did not result in detection of functional candidate gene(s) for red rot resistance, as the analysis was carried out on a single sugarcane genotype without studying the segregation pattern and epistatic interactions. Singh et al. [43] identified putative candidate genes for red rot resistance following linkage disequilibrium based association mapping; however, their involvement in imparting disease resistance has not been validated, thus restricting their use as molecular markers for identifying resistant genotypes and marker-aided selection in sugarcane.

The sucrose in sugarcane is synthesized in leaves and translocated via phloem to stalk where it is accumulated in parenchyma cells [44]. In the present study, sucrose accumulating stalk parenchyma cells of red rot susceptible sugarcane showed occurrence of fungal hyphae and loss of sucrose; the stored sucrose is proposed to be hydrolysed by *C*. *falcatum* causing red rot [45]. The cells of resistant transgenic plants were intact with no sucrose loss, due to inhibition of fungal hyphae to trail or swelling, lysis of hyphae. The hyphal lysis is due to the action of β-1,3-glucanase enzyme on β-1, 3-glucosyl linkages of fungal cell wall [12, 15]. The role of cell wall-degrading enzyme, β-1,3-glucanase in restricting *Colletotrichum* growth and activity has been demonstrated by Sun et al. [16]. The encoding gene *β-1*,*3-glucanase* belongs to pectate lyase superfamily protein pfam12708, carrying pectate lyase 3 catalytic domain; active site residues Glutamate 628 and Aspartate 569 of the catalytic domain acted as proton donor and nucleophile that have role in cleaving β-1,3-glycosidic bonds [46] and lysing *C*. *falcatum* hyphae leading to red rot resistance.

## Conclusions

Red rot resistant transgenic sugarcane was developed through expression of *β-1*,*3-glucanase* gene from *Trichoderma* spp. The transgenic plants with high transgene expression were resistant to two virulent pathotypes of *C*. *falcatum*, and displayed inhibition and/or lysis of *C*. *falcatum* hyphae in the sucrose-filled stalk parenchyma cells. The transgene expression was up-regulated after infection in the resistant plants. β-1,3-glucanase protein active site residues involved in cleaving β-1,3-glycosidic bonds leading to pathogen hyphal lysis were identified.

## Supporting information

S1 File Fig A. Amplification efficiency of *β-tubulin* gene in qRT-PCR determined by plotting cDNA dilution against C_T_ value. Fig B. Amplification efficiency of *β-1*,*3-glucanase* transgene in qRT-PCR determined by plotting cDNA dilution against C_T_ value. Fig C. Amplification chart of *β-tubulin* and *β-1*,*3-glucanase* genes obtained from qRT-PCR analysis. Fig D. Melt curve peaks of *β-tubulin* and *β-1*,*3-glucanase* genes obtained from qRT-PCR analysis. Fig E. Amplification chart from qRT-PCR analysis in leaves of CG_1_ transgenic plants before inoculation. Fig F. Melt curve peaks from qRT-PCR analysis in leaves of CG_1_ transgenic plants before inoculation. Fig G. Amplification chart from qRT-PCR analysis in roots of CG_1_ transgenic plants before inoculation.Fig H. Melt curve peaks from qRT-PCR analysis in roots of CG_1_ transgenic plants before inoculation. Fig I. Amplification chart from qRT-PCR analysis in leaves and roots of CG_1_ transgenic plants after inoculation. Fig J. Melt curve peaks from qRT-PCR analysis in leaves and roots of CG_1_ transgenic plants after inoculation.Fig K. Amplification chart obtained from qRT-PCR analysis of CG_2_ plants of SN10-12. Fig L. Melt curve peaks obtained from qRT-PCR analysis of CG_2_ plants of SN10-12. Fig M. Amplification chart obtained from qRT-PCR analysis of CG_2_ plants of SN10-13. Fig N. Melt curve peaks obtained from qRT-PCR analysis of CG_2_ plants of SN10-13. Fig O. PCR analysis of putative 127 CG_1_ plants raised through billet planting. Fig P. RNA integrity of putative PCR positive CG_1_ plants. Fig Q. PCR analysis of CG_2_ plants. Fig R. RNA integrity from 15 PCR positive CG_2_ plants. Fig S. Semi-quantitative RT-PCR analysis of CG_2_ plants with *26S rRNA* specific primers. Fig T. Semi-quantitative RT-PCR analysis of CG_2_ plants with *β-1*,*3-glucanase* transgene specific primers. Table A. The C_T_ values across replicates for six RT-PCR positive CG_1_ plants. Table B. The C_T_ values across replicates in leaves of CG_1_ transgenic plants before inoculation with Cf 09 pathotype. Table C. The C_T_ values across replicates in leaves of CG_1_ transgenic plants after inoculation with Cf 09 pathotype. Table D. The C_T_ values across replicates in roots of CG_1_ transgenic plants before inoculation with Cf 09 pathotype. Table E. The C_T_ values across replicates in roots of CG_1_ transgenic plants after inoculation with Cf 09 pathotype. Table F. The C_T_ values across replicates obtained for seven SN10-12 CG_2_ plants. Table G. The C_T_ values across replicates obtained for eight SN10-13 CG_2_ plants. Table H. Clonal propagation of putative T_0_ plants for raising CG_1_, and PCR analysis of CG_1_ plants. Table I. Relative *β-1*,*3-glucanase* expression in RT-PCR positive CG_1_ plants. Table J. Comparison of relative *β-1*,*3-glucanase* expression in leaves and roots of CG_1_ transgenic plants before and after inoculation with Cf 09 pathotype. Table K. Clonal propagation of red rot resistant CG_1_ transgenic plants for raising CG_2_, and PCR analysis of CG_2_ plants. Table L. Relative *β-1*,*3-glucanase* expression in CG_2_ plants. Table M. Identification of conserved active site residues in the catalytic domain of the protein.
(PDF)
