# Supplementary material for: Red rot resistant transgenic sugarcane developed through expression of β-1,3-glucanase gene
Source: PLoS One. 2017 Jun 28;12(6):e0179723. doi: 10.1371/journal.pone.0179723 (PMC5489175; doi:10.1371/journal.pone.0179723)
Supplement: S1 File — Fig A. Amplification efficiency of β-tubulin gene in qRT-PCR determined by plotting cDNA dilution against CT value. Fig B. Amplification efficiency of β-1,3-glucanase transgene in qRT-PCR determined by plotting cDNA dilution against CT value. Fig C. Amplification chart of β-tubulin and β-1,3-glucanase genes obtained from qRT-PCR analysis. Fig D. Melt curve peaks of β-tubulin and β-1,3-glucanase genes obtained from qRT-PCR analysis. Fig E. Amplification chart from qRT-PCR analysis in leaves of CG1 transgenic plants before inoculation. Fig F. Melt curve peaks from qRT-PCR analysis in leaves of CG1 transgenic plants before inoculation. Fig G. Amplification chart from qRT-PCR analysis in roots of CG1 transgenic plants before inoculation.Fig H. Melt curve peaks from qRT-PCR analysis in roots of CG1 transgenic plants before inoculation. Fig I. Amplification chart from qRT-PCR analysis in leaves and roots of CG1 transgenic plants after inoculation. Fig J. Melt curve peaks from qRT-PCR analysis in leaves and roots of CG1 transgenic plants after inoculation.Fig K. Amplification chart obtained from qRT-PCR analysis of CG2 plants of SN10-12. Fig L. Melt curve peaks obtained from qRT-PCR analysis of CG2 plants of SN10-12. Fig M. Amplification chart obtained from qRT-PCR analysis of CG2 plants of SN10-13. Fig N. Melt curve peaks obtained from qRT-PCR analysis of CG2 plants of SN10-13. Fig O. PCR analysis of putative 127 CG1 plants raised through billet planting. Fig P. RNA integrity of putative PCR positive CG1 plants. Fig Q. PCR analysis of CG2 plants. Fig R. RNA integrity from 15 PCR positive CG2 plants. Fig S. Semi-quantitative RT-PCR analysis of CG2 plants with 26S rRNA specific primers. Fig T. Semi-quantitative RT-PCR analysis of CG2 plants with β-1,3-glucanase transgene specific primers. Table A. The CT values across replicates for six RT-PCR positive CG1 plants. Table B. The CT values across replicates in leaves of CG1 transgenic plants before inoculation with Cf 09 patho [file pone.0179723.s001.pdf]

Supporting Information (S1 file)

Red rot resistant transgenic sugarcane developed through expression of *β-1,3-glucanase* gene

Shivani Nayyar[1] • Bipen Kumar Sharma[2] • Ajinder Kaur[1] • Anu Kalia[3] • Gulzar Singh Sanghera[2] • Karanjit Singh Thind[4] • Inderjit Singh Yadav[1] • Jagdeep Singh Sandhu[1*]

[1]School of Agricultural Biotechnology
Punjab Agricultural University, Ludhiana 141 004, India

[2]Punjab Agricultural University
Regional Research Station, Kapurthala 144 601, India

[3]Electron Microscopy and Nanoscience Laboratory
Punjab Agricultural University, Ludhiana 141 004, India

[4]Department of Plant Breeding and Genetics
Punjab Agricultural University, Ludhiana 141 004, India

∗ Corresponding author
js_sandhu@pau.edu

Fig A. Amplification efficiency of *β-tubulin* gene in qRT-PCR determined by plotting cDNA dilution against C_T value.

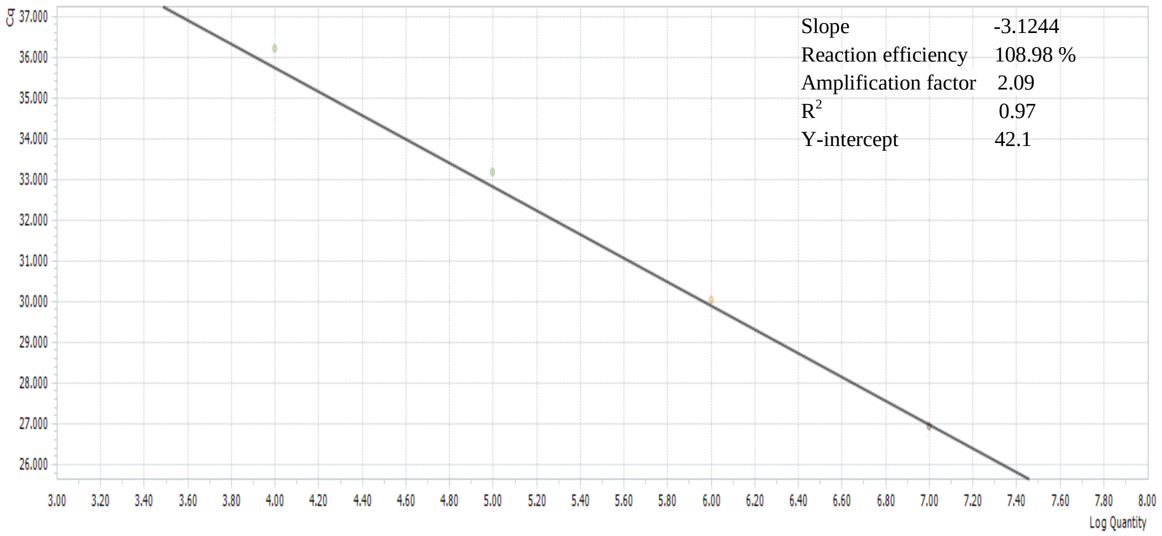

Fig B. Amplification efficiency of *β-1,3-glucanase* transgene in qRT-PCR determined by plotting cDNA dilution against C_T value.

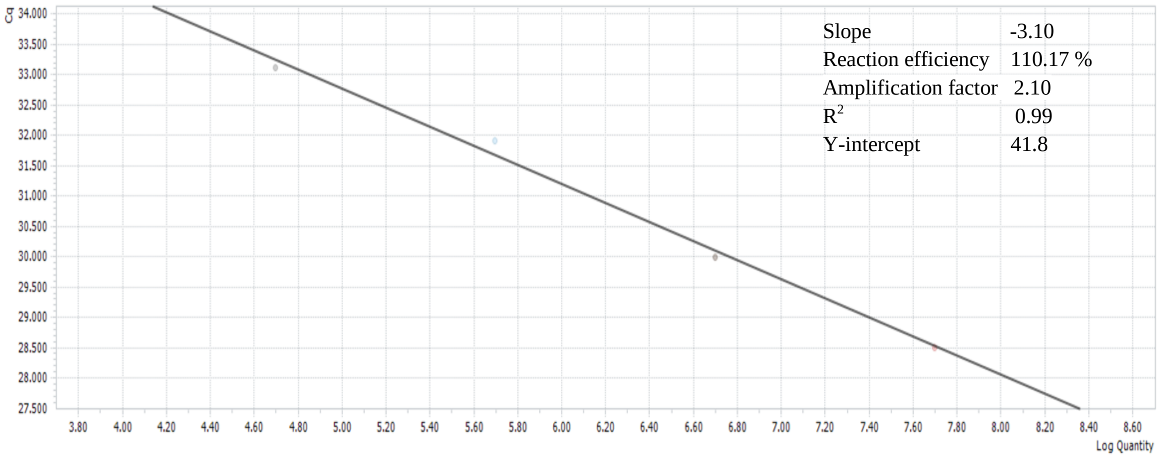

Fig C. Amplification chart of *β-tubulin* and *β-1,3-glucanase* genes obtained from qRT-PCR analysis.

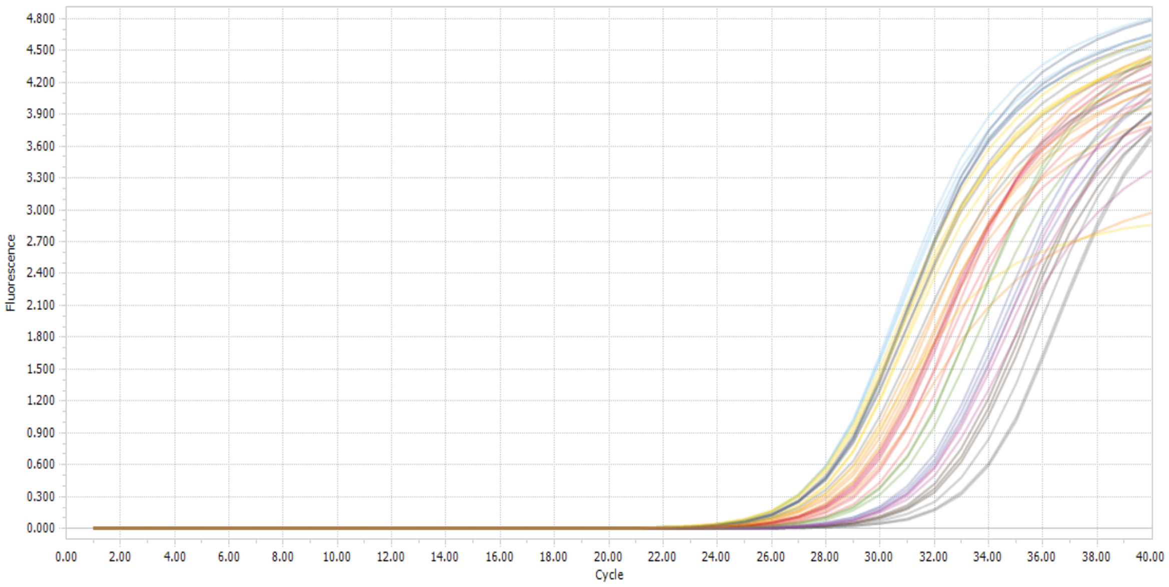

Fig D. Melt curve peaks of *β-tubulin* and *β-1,3-glucanase* genes obtained from qRT-PCR analysis.

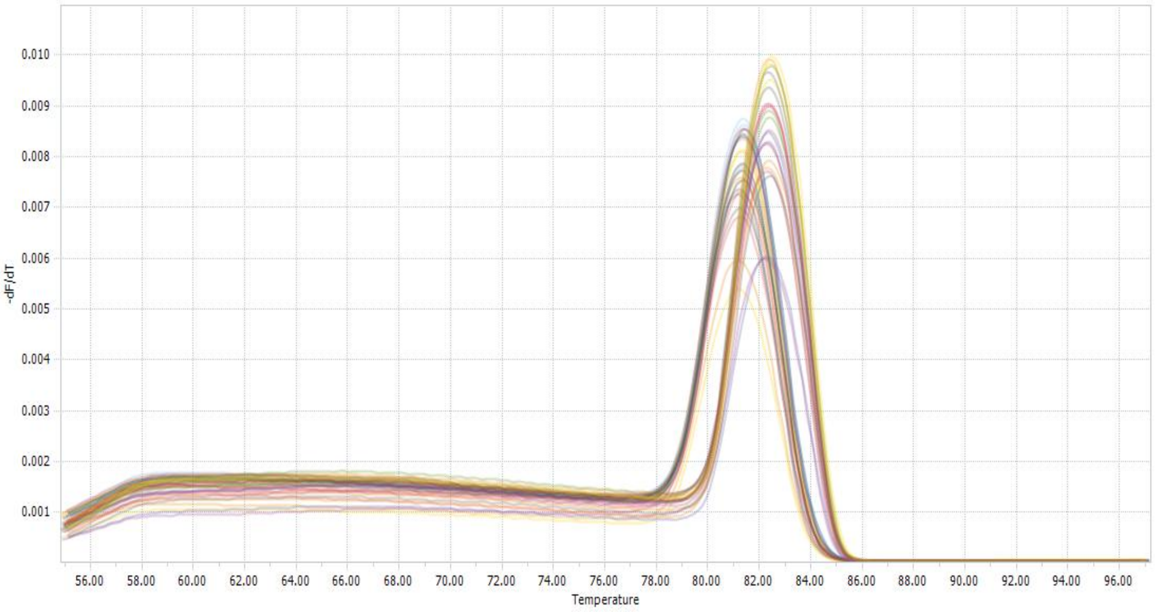

Fig E. Amplification chart from qRT-PCR analysis in leaves of CG_1 transgenic plants before inoculation.

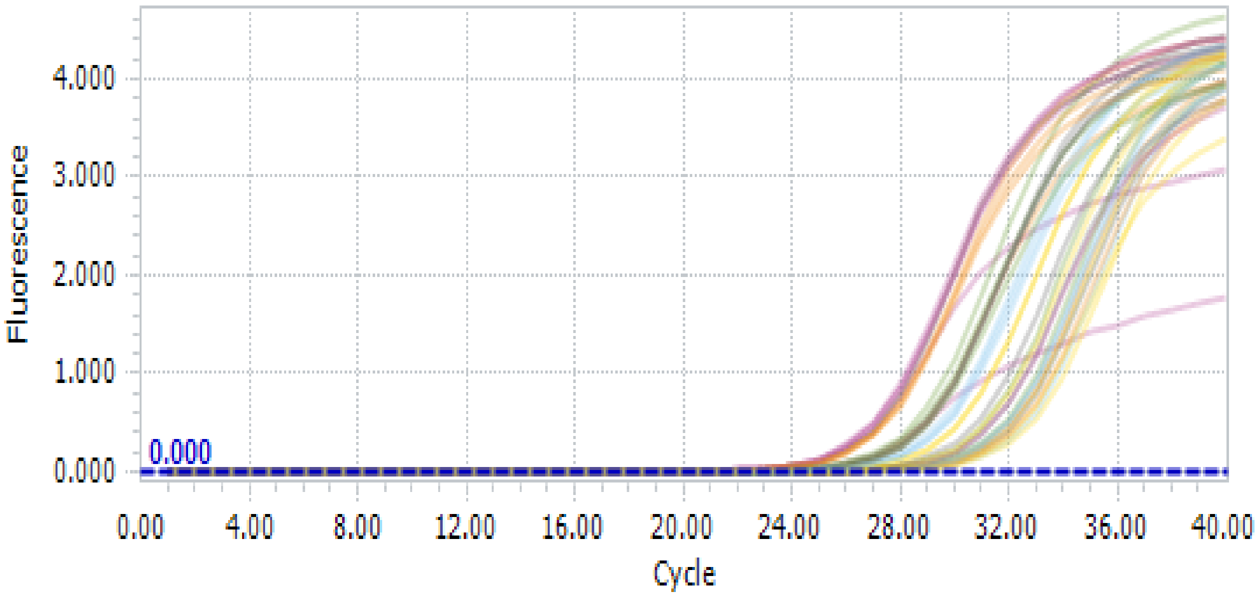

Fig F. Melt curve peaks from qRT-PCR analysis in leaves of CG_1 transgenic plants before inoculation.

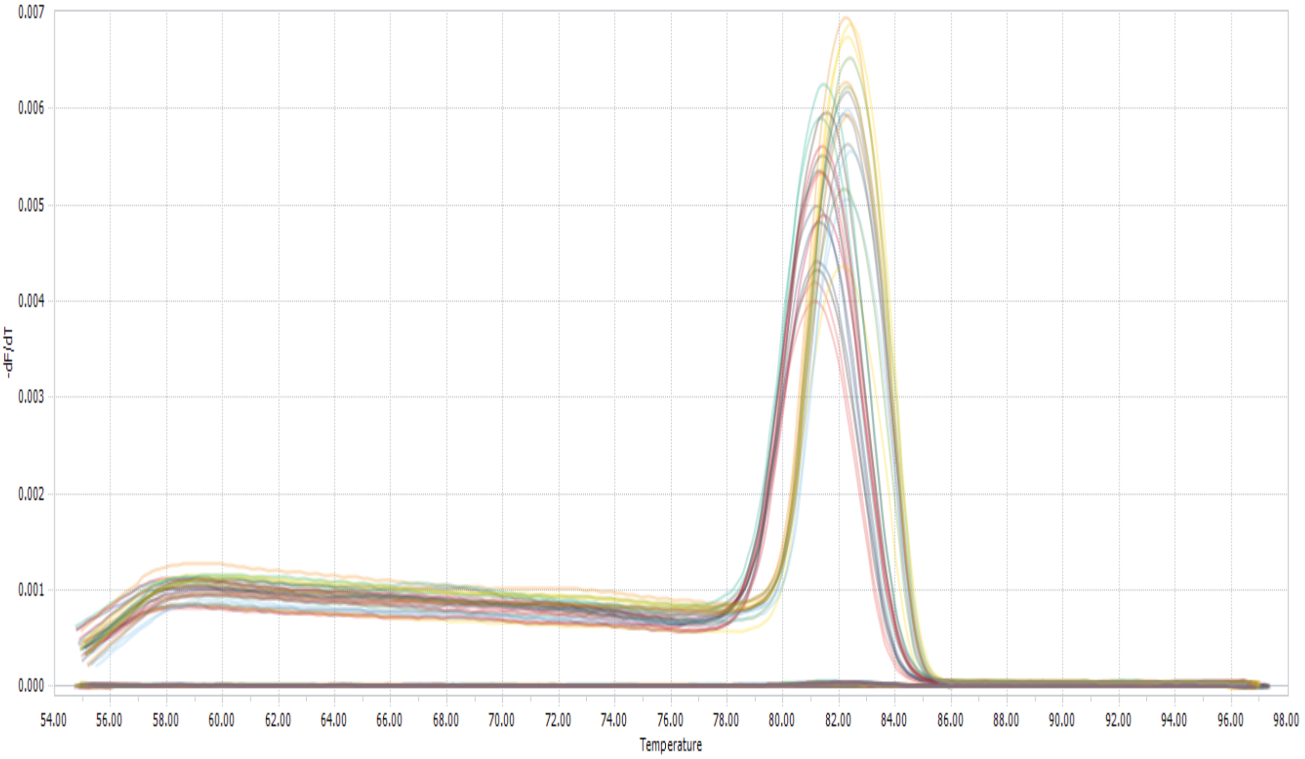

Fig G. Amplification chart from qRT-PCR analysis in roots of CG_1 transgenic plants before inoculation.

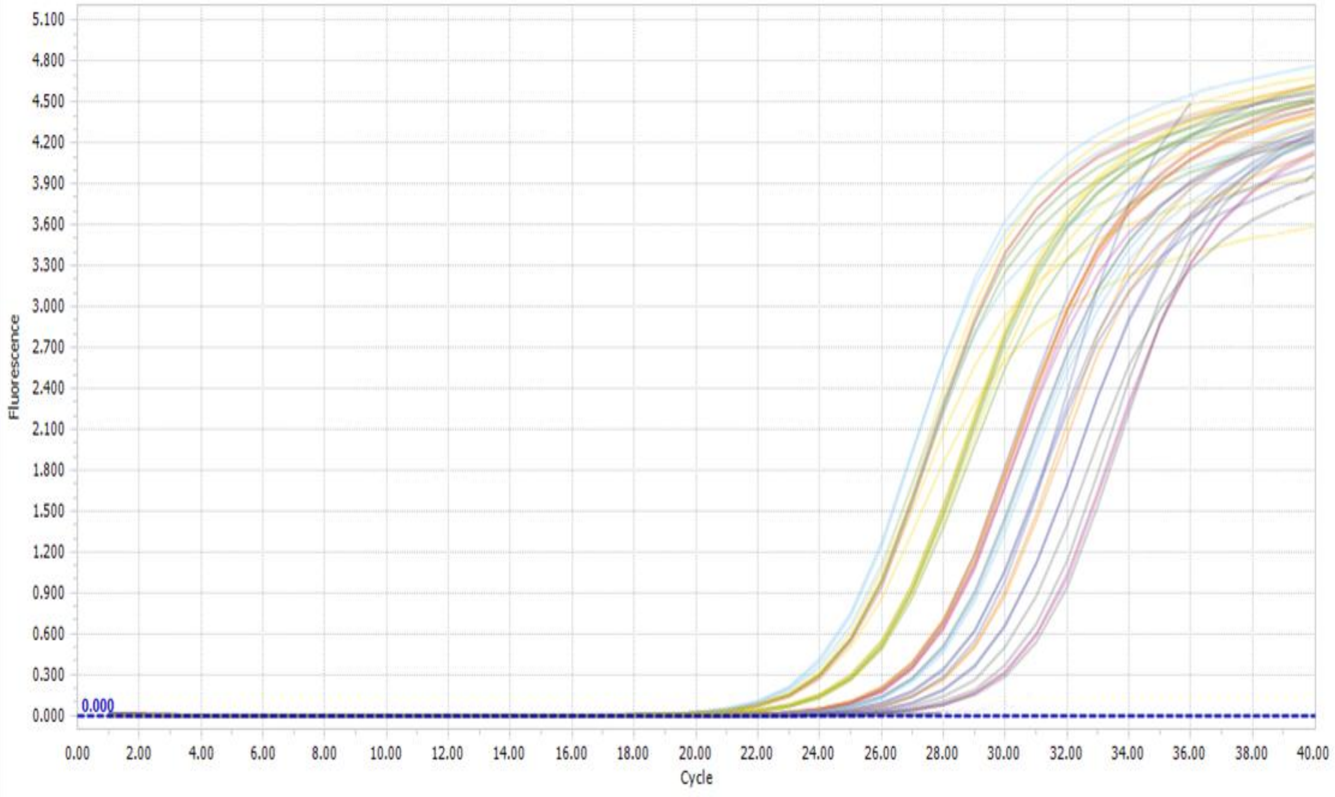

Fig H. Melt curve peaks from qRT-PCR analysis in roots of CG_1 transgenic plants before inoculation.

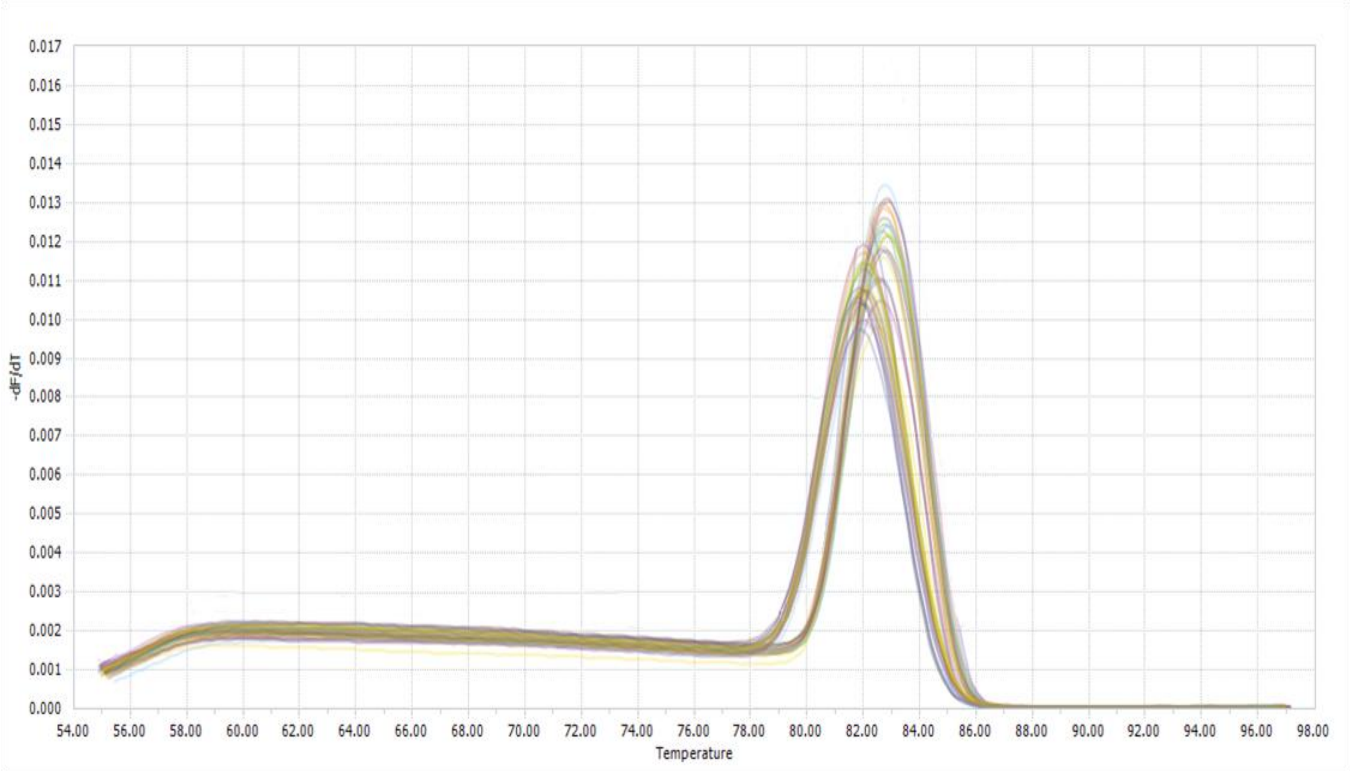

Fig I. Amplification chart from qRT-PCR analysis in leaves and roots of CG_1 transgenic plants after inoculation.

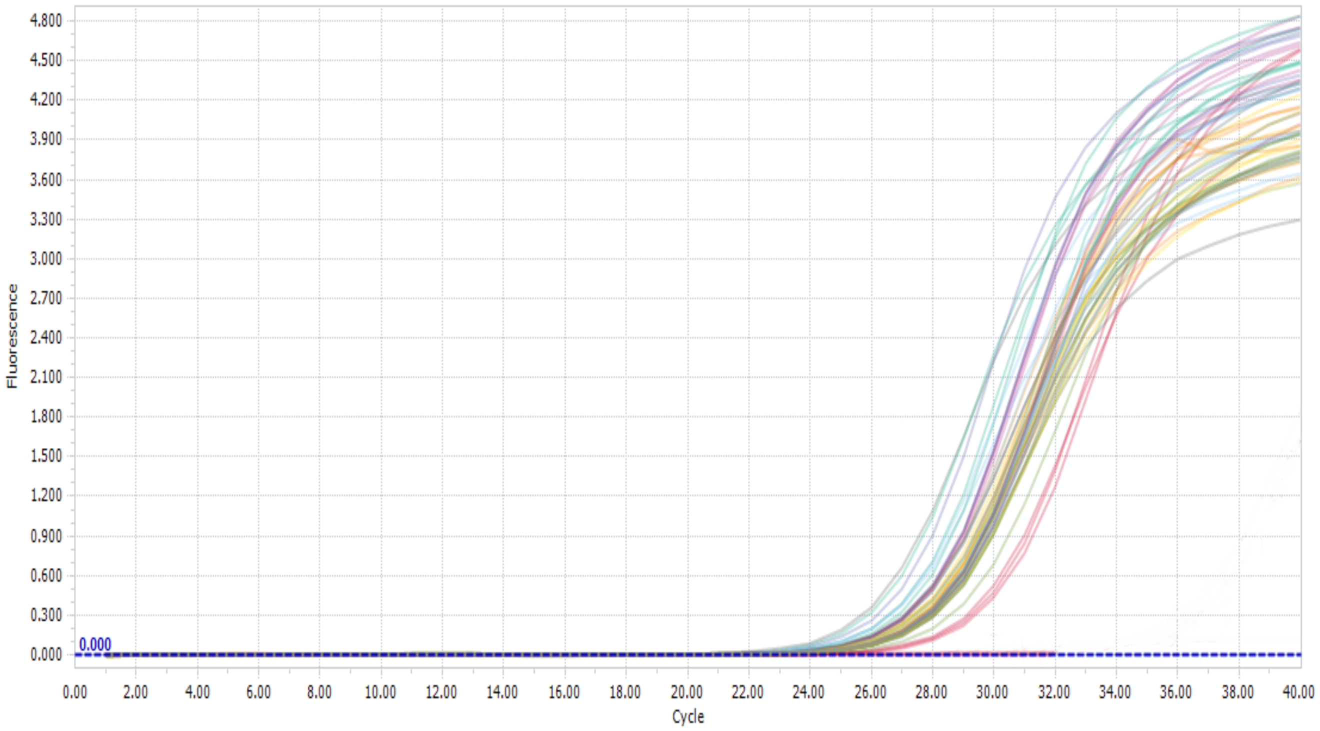

Fig J. Melt curve peaks from qRT-PCR analysis in leaves and roots of CG_1 transgenic plants after inoculation.

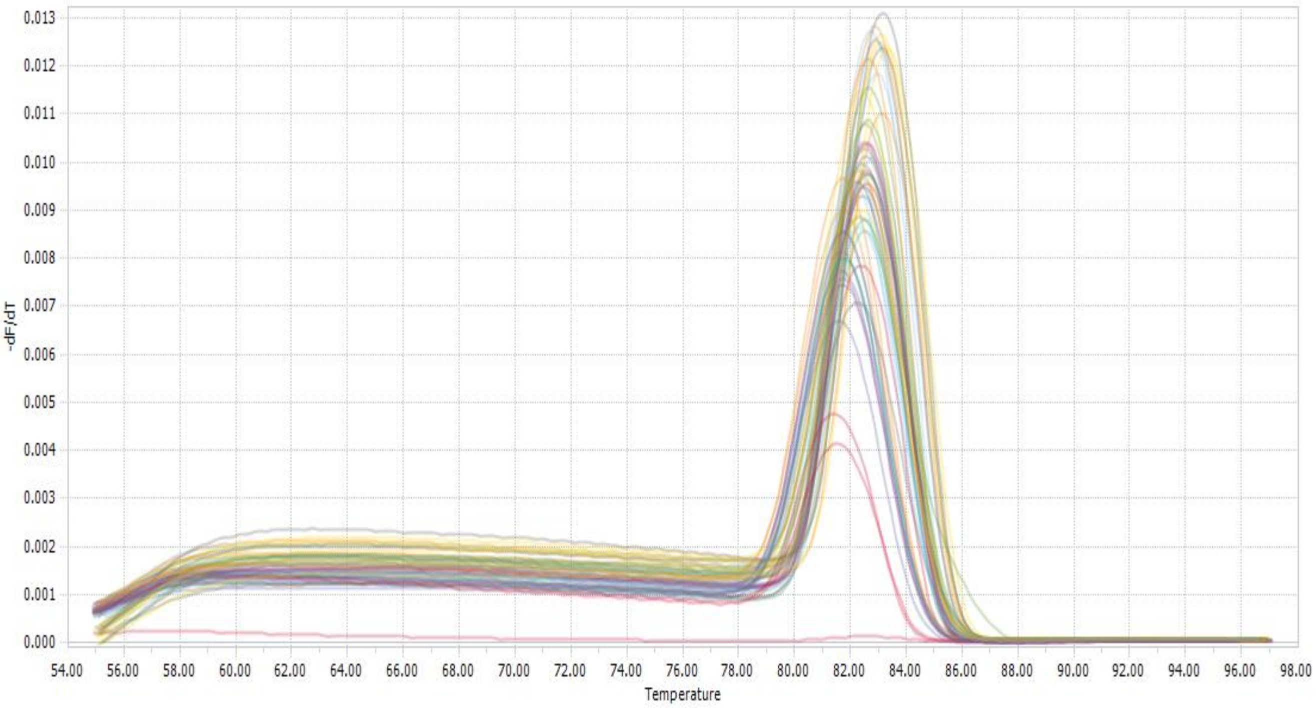

Fig K. Amplification chart obtained from qRT-PCR analysis of CG_2 plants of SN10-12.

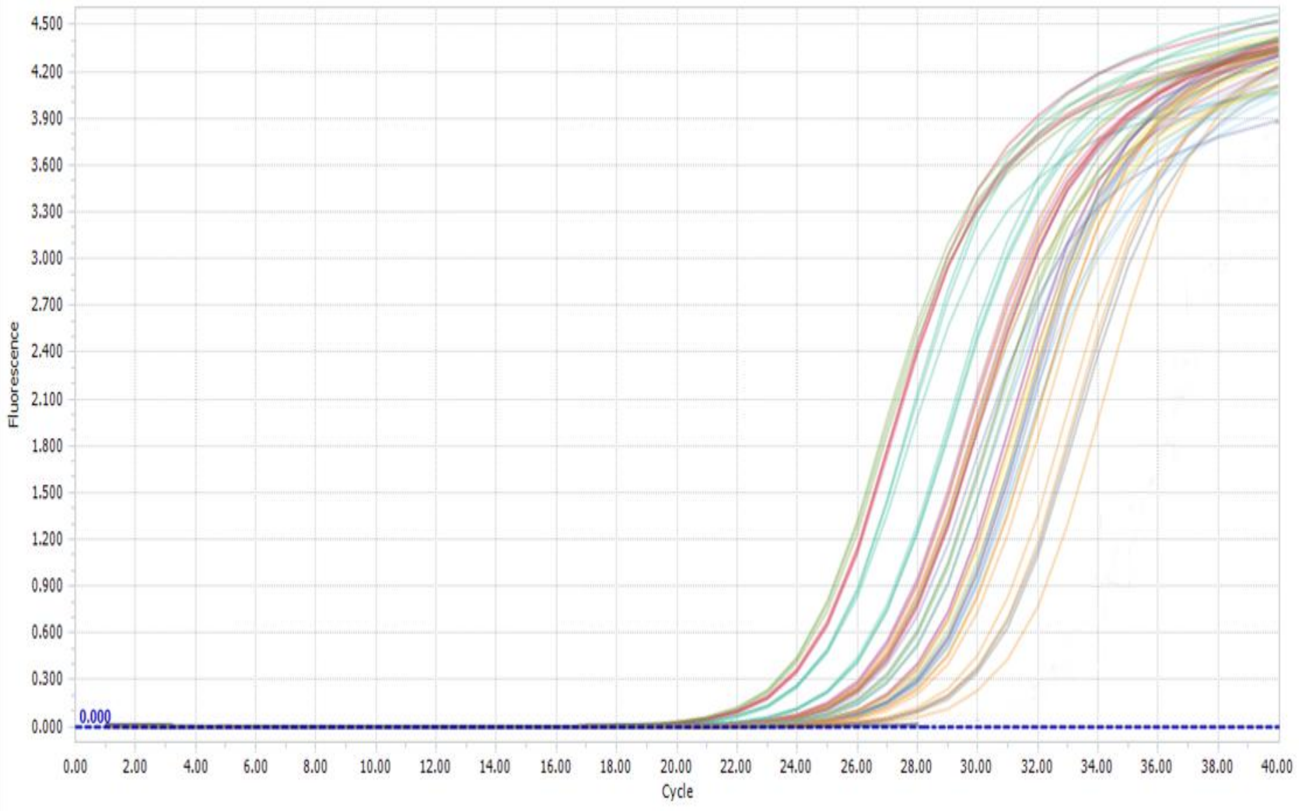

Fig L. Melt curve peaks obtained from qRT-PCR analysis of CG_2 plants of SN10-12.

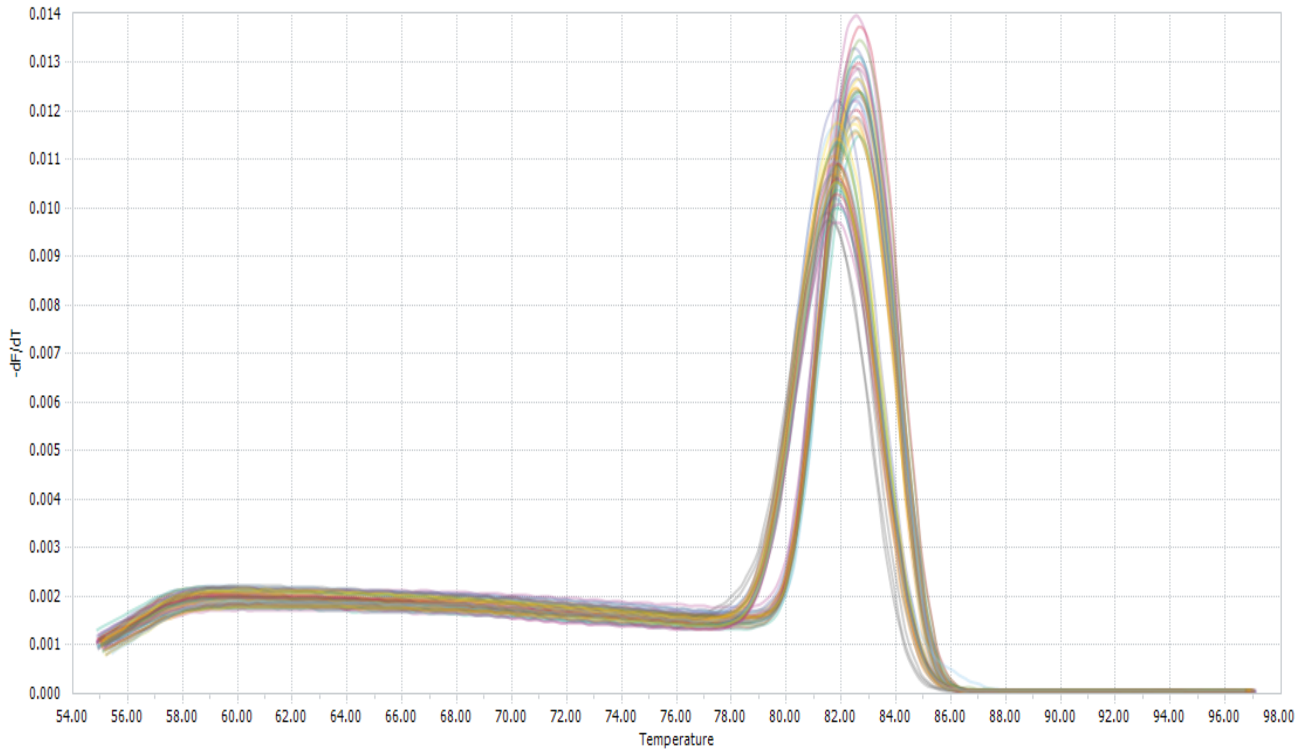

Fig M. Amplification chart obtained from qRT-PCR analysis of CG_2 plants of SN10-13.

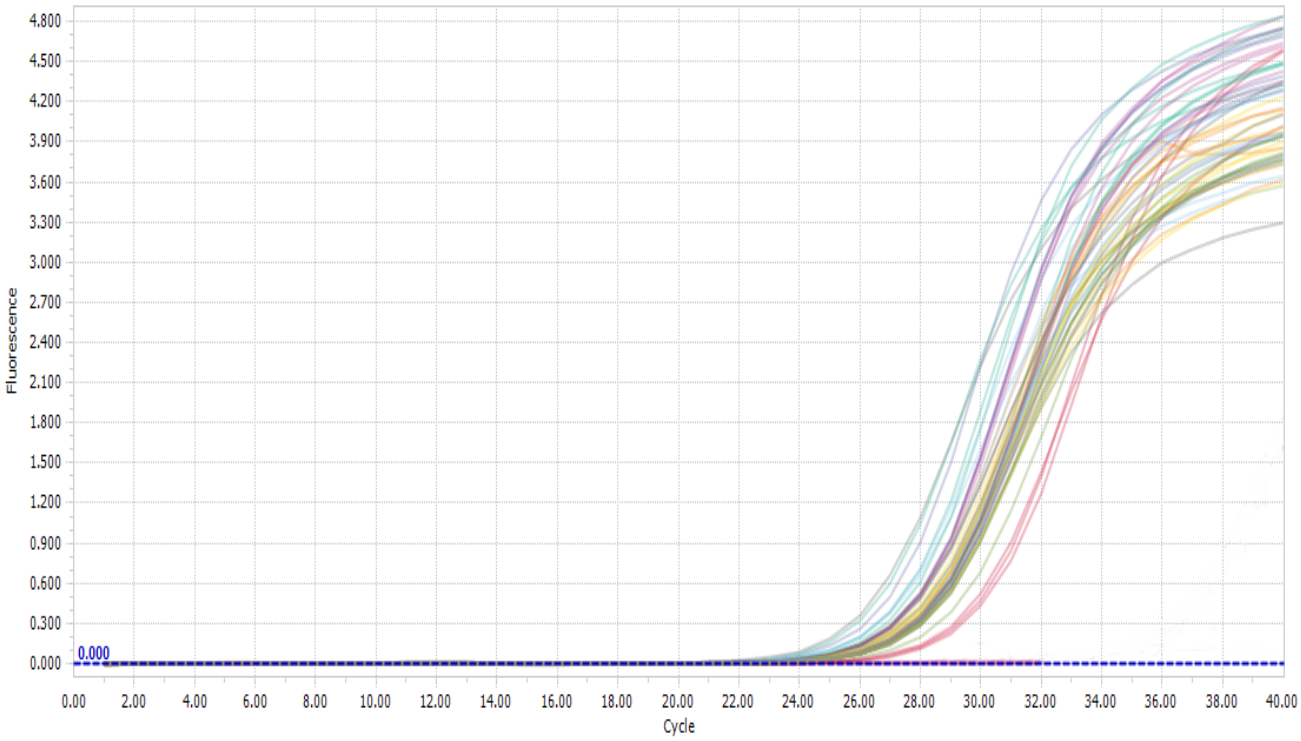

Fig N. Melt curve peaks obtained from qRT-PCR analysis of CG_2 plants of SN10-13.

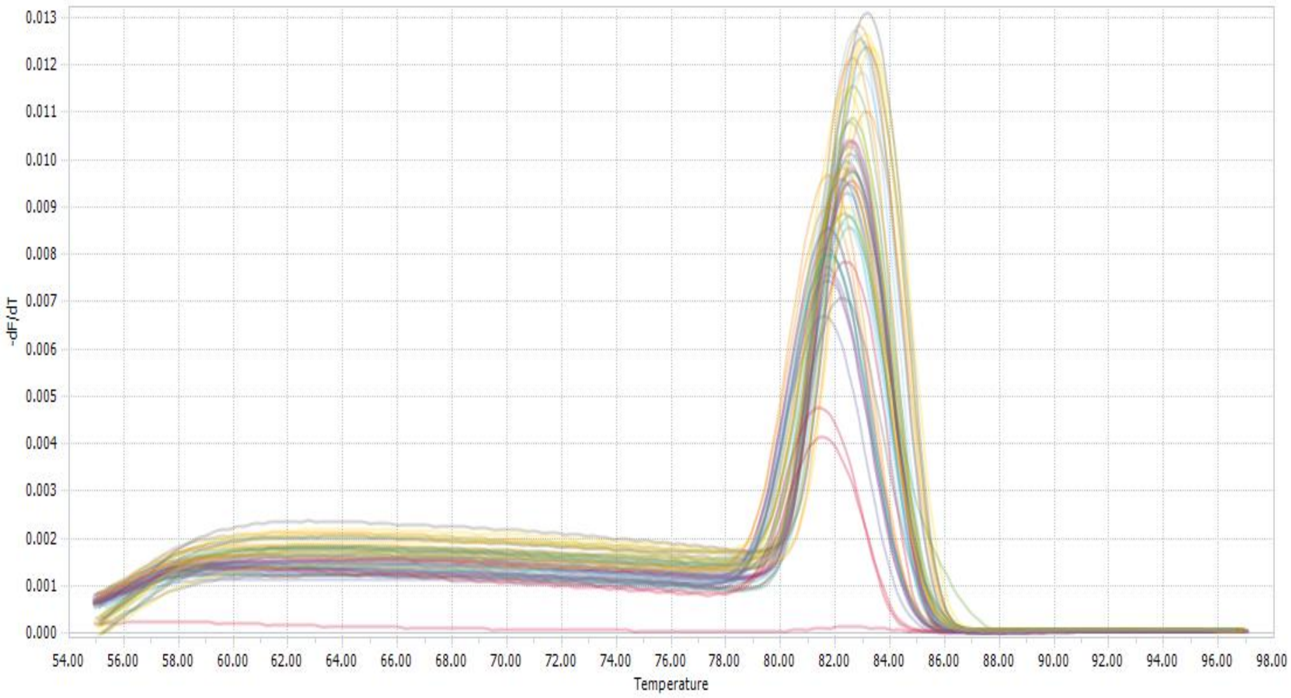

Fig O. PCR analysis of putative 127 CG_1 plants raised through billet planting. L refers to 1 Kb ladder (Promega, Cat. No. G5711); WC Water Control; PC Plasmid Control; SN1-1 to SN10-26 represent putative CG_1 plants; NC Negative Control from non-transgenic plant.

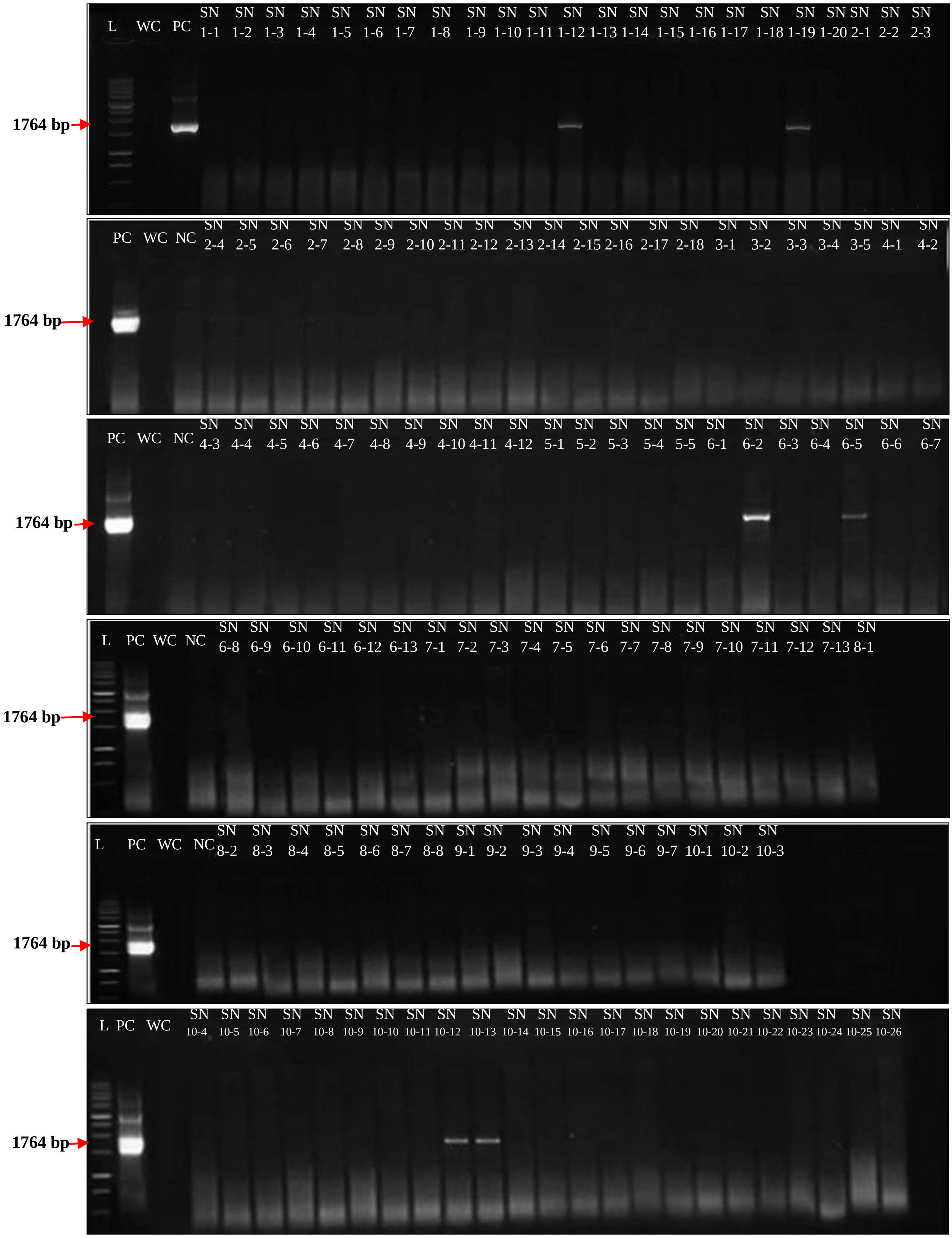

Fig P. RNA integrity of putative PCR positive CG_1 plants. The numbers SN10-12, SN10-13, SN6-2, SN6-5, SN1-12 and SN1-19 refer to RNA isolated from six putative PCR positive CG_1 plants; NC1 and NC2 refer to RNA isolated from the Negative Controls (non-transgenic plants).

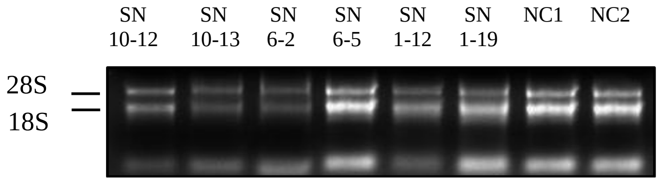

Fig Q. PCR analysis of CG_2 plants. L refers to 1 Kb ladder (Promega, Cat. No. G5711); PC Plasmid Control; WC Water Control; C1-1 to C1-7 are CG_2 clonal plants of transgenic plant SN10-12 and C2-1 to C2-8 are CG_2 clonal plants of transgenic plant SN10-13; B Blank well.

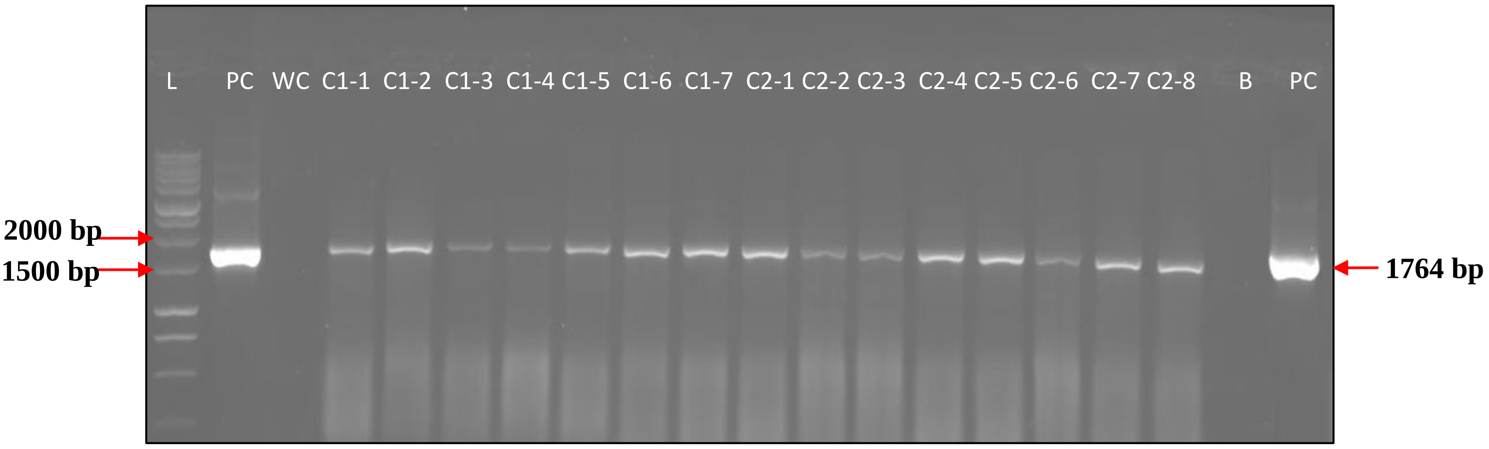

Fig R. RNA integrity from 15 PCR positive CG_2 plants. The numbers C1-1 to C1-7 are CG_2 clonal plants of SN10-12 and C2-1 to C2-8 are CG_2 clonal plants of SN10-13.

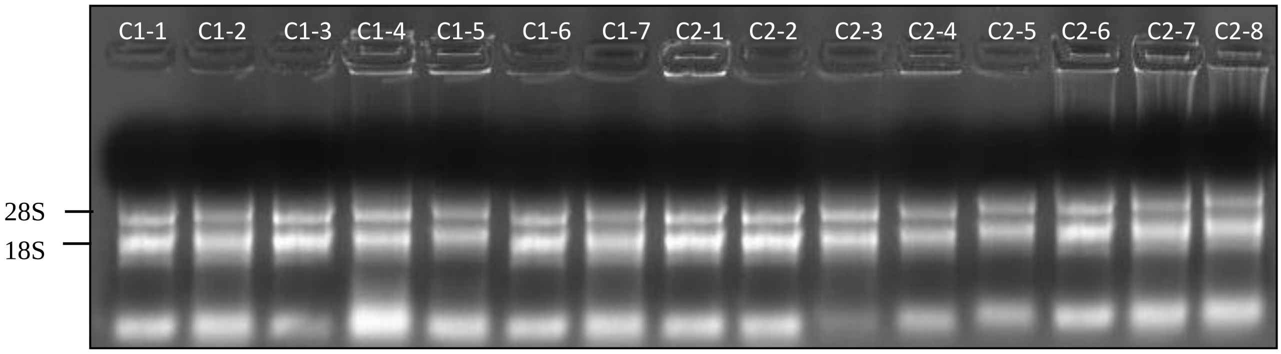

Fig S. Semi-quantitative RT-PCR analysis of CG_2 plants with *26S rRNA* specific primers. C1-1 to C-7 and C2-1 to C2-8 represent amplification of *26S rRNA* gene in 15 PCR positive CG_2 plants; L refers to 100 bp ladder (Takara, Cat. No. 3407A).

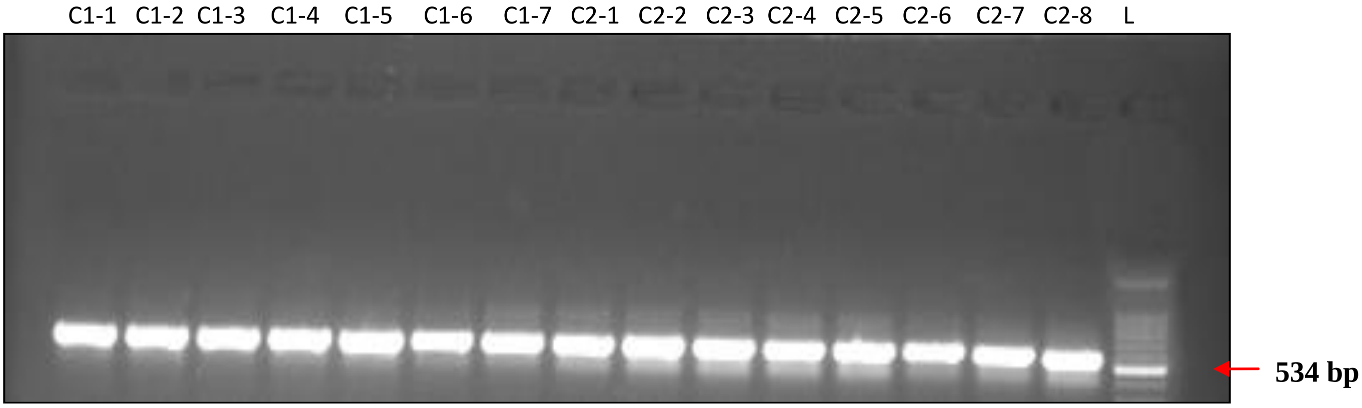

Fig T. Semi-quantitative RT-PCR analysis of CG_2 plants with *β-1,3-glucanase* transgene specific primers. C1-1 to C1-7 and C2-1 to C2-8 represent amplification of transgene in 15 PCR positive CG_2 plants; L refers to 1 Kb ladder (Promega, Cat. No. G5711).

C2-1 C2-2 C2-3 C2-4 C2-5 C2-6 C2-7 C2-8 L C1-1 C1-2 C1-3 C1-4 C1-5 C1-6 C1-7

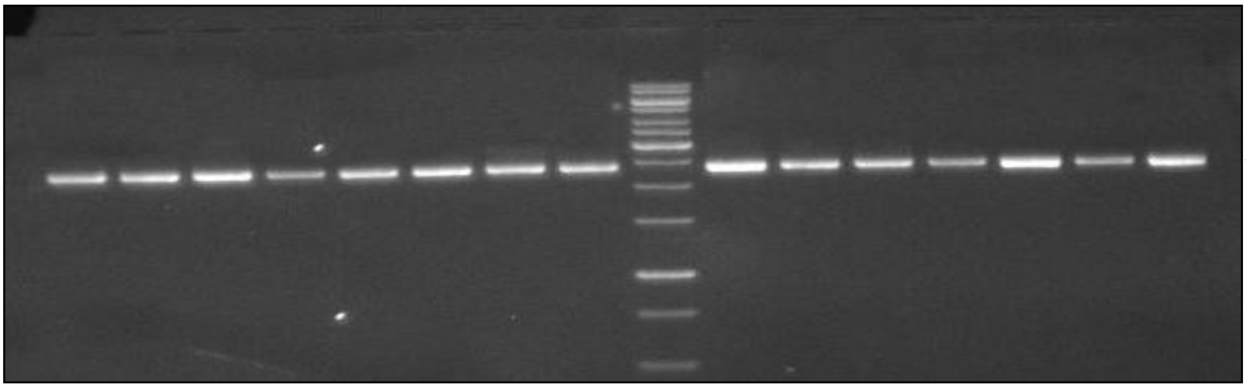

1764 bp

Table A. The C_T values across replicates for six RT-PCR positive CG_1 plants.

Plant number	Replicate	Gene	Threshold cycle (C_T)	C_T mean
Non-transgenic	1	*β-tubulin*	27.22	27.02
	1		26.93	
	1		26.92	
	1	*β-1,3-glucanase*	28.05	27.96
	1		27.87	
	1		27.95	
SN1-12	2	*β-tubulin*	26.31	26.33
	2		26.32	
	2		26.35	
	2	*β-1,3-glucanase*	26.25	26.31
	2		26.38	
	2		26.31	
SN1-19	3	*β-tubulin*	31.64	31.69
	3		31.68	
	3		31.76	
	3	*β-1,3-glucanase*	30.86	30.66
	3		30.52	
	3		30.60	
SN6-2	4	*β-tubulin*	26.67	26.65
	4		26.59	
	4		26.70	
	4	*β-1,3-glucanase*	27.08	26.78
	4		26.67	
	4		26.60	
SN6-5	5	*β-tubulin*	28.14	27.91
	5		27.77	
	5		27.81	
	5	*β-1,3-glucanase*	30.25	30.09
	5		29.96	
	5		30.07	
SN10-12	6	*β-tubulin*	28.82	28.53
	6		28.46	
	6		28.32	
	6	*β-1,3-glucanase*	27.50	27.37
	6		27.40	
	6		27.20	
SN10-13	7	*β-tubulin*	30.75	30.31
	7		30.04	
	7		30.16	
	7	*β-1,3-glucanase*	29.97	29.10
	7		29.29	
	7		28.04	

Table B. The C_T values across replicates in leaves of CG_1 transgenic plants before inoculation with Cf 09 pathotype.

Plant number	Replicate	Gene	Threshold cycle (C_T)	C_T mean
Non-transgenic	1	*β-tubulin*	27.51	27.49
	1		27.49	
	1		27.49	
	1	*β-1,3-glucanase*	27.39	27.40
	1		28.41	
	1		27.40	
SN1-12	2	*β-tubulin*	30.67	30.67
	2		30.66	
	2		30.67	
	2	*β-1,3-glucanase*	29.56	29.57
	2		29.57	
	2		29.57	
SN1-19	3	*β-tubulin*	29.86	29.87
	3		29.87	
	3		29.87	
	3	*β-1,3-glucanase*	27.86	27.86
	3		27.85	
	3		27.86	
SN6-2	4	*β-tubulin*	30.04	30.05
	4		30.05	
	4		30.05	
	4	*β-1,3-glucanase*	29.30	29.29
	4		29.29	
	4		29.29	
SN6-5	5	*β-tubulin*	27.16	27.16
	5		27.15	
	5		27.16	
	5	*β-1,3-glucanase*	28.42	28.42
	5		28.42	
	5		28.41	
SN10-12	6	*β-tubulin*	29.35	29.35
	6		29.35	
	6		29.34	
	6	*β-1,3-glucanase*	27.24	27.25
	6		27.25	
	6		27.25	
SN10-13	7	*β-tubulin*	29.44	29.43
	7		29.43	
	7		29.44	
	7	*β-1,3-glucanase*	27.24	27.25
	7		27.25	
	7		27.25	

Table C. The C_T values across replicates in leaves of CG_1 transgenic plants after inoculation with Cf 09 pathotype.

Plant number	Replicate	Gene	Threshold cycle (C_T)	C_T mean
Non-transgenic	1	*β-tubulin*	25.38	25.39
	1		25.39	
	1		25.39	
	1	*β-1,3-glucanase*	25.44	25.42
	1		25.42	
	1		25.41	
SN1-12	2	*β-tubulin*	27.76	27.77
	2		27.76	
	2		27.77	
	2	*β-1,3-glucanase*	26.54	26.55
	2		26.56	
	2		26.54	
SN1-19	3	*β-tubulin*	29.95	29.95
	3		29.94	
	3		29.95	
	3	*β-1,3-glucanase*	28.01	28.01
	3		28.02	
	3		28.01	
SN6-2	4	*β-tubulin*	29.21	29.22
	4		29.22	
	4		29.22	
	4	*β-1,3-glucanase*	28.37	28.37
	4		28.36	
	4		28.36	
SN6-5	5	*β-tubulin*	27.94	27.95
	5		27.96	
	5		27.94	
	5	*β-1,3-glucanase*	30.74	30.75
	5		30.75	
	5		30.74	
SN10-12	6	*β-tubulin*	26.07	26.07
	6		26.06	
	6		26.07	
	6	*β-1,3-glucanase*	29.276	29.276
	6		29.275	
	6		29.275	
SN10-13	7	*β-tubulin*	28.92	28.93
	7		28.93	
	7		28.93	
	7	*β-1,3-glucanase*	26.06	26.07
	7		26.07	
	7		26.07	

Table D. The C_T values across replicates in roots of CG_1 transgenic plants before inoculation with Cf 09 pathotype.

Plant number	Replicate	Gene	Threshold cycle (C_T)	C_T mean
Non-transgenic	1	*β-tubulin*	26.81	26.83
	1		26.84	
	1		26.84	
	1	*β-1,3-glucanase*	26.88	26.90
	1		26.92	
	1		26.91	
SN1-12	2	*β-tubulin*	24.81	24.82
	2		24.83	
	2		24.83	
	2	*β-1,3-glucanase*	24.80	24.79
	2		24.81	
	2		24.78	
SN1-19	3	*β-tubulin*	27.41	27.42
	3		27.43	
	3		27.43	
	3	*β-1,3-glucanase*	26.80	26.82
	3		26.84	
	3		26.81	
SN6-2	4	*β-tubulin*	26.03	26.04
	4		26.05	
	4		26.01	
	4	*β-1,3-glucanase*	26.18	26.21
	4		26.20	
	4		26.22	
SN6-5	5	*β-tubulin*	25.52	25.52
	5		25.50	
	5		25.51	
	5	*β-1,3-glucanase*	26.01	26.02
	5		26.02	
	5		26.02	
SN10-12	6	*β-tubulin*	24.47	24.48
	6		24.48	
	6		24.48	
	6	*β-1,3-glucanase*	23.78	23.80
	6		23.81	
	6		23.81	
SN10-13	7	*β-tubulin*	24.58	24.60
	7		24.61	
	7		24.61	
	7	*β-1,3-glucanase*	23.78	23.80
	7		23.81	
	7		23.81	

Table E. The C_T values across replicates in roots of CG_1 transgenic plants after inoculation with Cf 09 pathotype.

Plant number	Replicate	Gene	Threshold cycle (C_T)	C_T mean
Non-transgenic	1	*β-tubulin*	25.40	25.42
	1		25.40	
	1		25.44	
	1	*β-1,3-glucanase*	26.01	26.02
	1		26.05	
	1		26.02	
SN1-12	2	*β-tubulin*	25.80	25.79
	2		25.81	
	2		25.78	
	2	*β-1,3-glucanase*	26.04	26.04
	2		26.03	
	2		26.04	
SN1-19	3	*β-tubulin*	25.14	25.14
	3		25.13	
	3		25.14	
	3	*β-1,3-glucanase*	25.04	25.04
	3		25.04	
	3		25.03	
SN6-2	4	*β-tubulin*	28.67	28.67
	4		28.65	
	4		28.67	
	4	*β-1,3-glucanase*	28.52	28.56
	4		28.56	
	4		28.57	
SN6-5	5	*β-tubulin*	29.94	29.95
	5		29.94	
	5		29.95	
	5	*β-1,3-glucanase*	30.70	30.75
	5		29.74	
	5		30.75	
SN10-12	6	*β-tubulin*	27.50	27.50
	6		27.49	
	6		27.50	
	6	*β-1,3-glucanase*	25.07	25.09
	6		25.10	
	6		25.09	
SN10-13	7	*β-tubulin*	27.05	27.06
	7		27.06	
	7		27.06	
	7	*β-1,3-glucanase*	25.07	25.09
	7		25.10	
	7		25.09	

Table F. The C_T values across replicates obtained for seven SN10-12 CG_2 plants.

Plant number	Replicate	Gene	Threshold cycle (C_T)	C_T mean
Non-transgenic	1	*β-tubulin*	26.75	26.83
	1		26.85	
	1		26.90	
	1	*β-1,3-glucanase*	27.86	27.90
	1		28.01	
	1		27.88	
C1-1	2	*β-tubulin*	25.96	26.02
	2		26.08	
	2		26.01	
	2	*β-1,3-glucanase*	25.80	25.82
	2		25.85	
	2		25.84	
C1-2	3	*β-tubulin*	29.80	29.79
	3		29.78	
	3		29.81	
	3	*β-1,3-glucanase*	26.61	29.60
	3		26.61	
	3		26.57	
C1-3	4	*β-tubulin*	24.78	24.82
	4		24.85	
	4		24.83	
	4	*β-1,3-glucanase*	24.52	24.52
	4		24.58	
	4		24.52	
C1-4	5	*β-tubulin*	27.38	27.42
	5		27.45	
	5		27.43	
	5	*β-1,3-glucanase*	26.90	26.92
	5		26.90	
	5		26.95	
C1-5	6	*β-tubulin*	27.89	26.52
	6		27.84	
	6		27.88	
	6	*β-1,3-glucanase*	26.48	26.08
	6		26.55	
	6		26.53	
C1-6	7	*β-tubulin*	24.54	24.54
	7		24.56	
	7		24.53	
	7	*β-1,3-glucanase*	24.26	24.25
	7		24.22	
	7		24.26	
C1-7	8	*β-tubulin*	24.82	24.80
	8		24.78	
	8		24.82	
	8	*β-1,3-glucanase*	24.62	24.60
	8		24.58	
	8		24.62	

Table G. The C_T values across replicates obtained for eight SN10-13 CG_2 plants.

Plant number	Replicate	Gene	Threshold cycle (C_T)	C_T mean
Non-transgenic	1	*β-tubulin*	27.22	27.16
	1		27.14	
	1		27.14	
	1	*β-1,3-glucanase*	26.14	26.66
	1		27.08	
	1		26.70	
C2-1	2	*β-tubulin*	27.81	27.80
	2		27.91	
	2		27.81	
	2	*β-1,3-glucanase*	26.06	26.06
	2		26.05	
	2		26.06	
C2-2	3	*β-tubulin*	27.83	27.84
	3		27.84	
	3		27.84	
	3	*β-1,3-glucanase*	26.14	26.15
	3		26.15	
	3		26.15	
C2-3	4	*β-tubulin*	27.92	27.94
	4		27.96	
	4		27.95	
	4	*β-1,3-glucanase*	26.10	26.14
	4		26.22	
	4		26.12	
C2-4	5	*β-tubulin*	28.01	27.81
	5		27.70	
	5		27.79	
	5	*β-1,3-glucanase*	25.96	26.02
	5		26.08	
	5		26.01	
C2-5	6	*β-tubulin*	27.89	27.87
	6		27.84	
	6		27.88	
	6	*β-1,3-glucanase*	26.04	26.08
	6		26.10	
	6		26.11	
C2-6	7	*β-tubulin*	27.92	27.90
	7		27.88	
	7		27.90	
	7	*β-1,3-glucanase*	26.03	26.01
	7		26.01	
	7		26.03	
C2-7	8	*β-tubulin*	27.99	27.96
	8		27.94	
	8		27.95	
	8	*β-1,3-glucanase*	26.20	26.17
	8		26.15	
	8		26.16	
C2-8	9	*β-tubulin*	27.97	27.96
	9		27.96	
	9		27.95	
	9	*β-1,3-glucanase*	26.06	26.06
	9		26.06	
	9		26.05	

Table H. Clonal propagation of putative T_0 plants for raising CG_1, and PCR analysis of CG_1 plants.

Putative T_0 plant	Number of billets planted	Number of billets sprouted	Designation of CG_1 plants	Number of PCR positive CG_1 plants	Designation of PCR positive plants
IP1	22	20	SN1-1 to SN1-20	2	SN1-12, SN1-19
IP2	20	18	SN2-1 to SN2-18	-	-
IP3	41	5	SN3-1 to SN3-5	-	-
IP4	22	12	SN4-1 to SN4-12	-	-
IP5	31	5	SN5-1 to SN5-5	-	-
IP6	38	13	SN6-1 to SN6-13	2	SN6-2, SN6-5
IP7	30	13	SN7-1 to SN7-13	-	-
IP8	20	8	SN8-1 to SN8-8	-	-
IP9	36	7	SN9-1 to SN9-7	-	-
IP10	35	26	SN10-1 to SN10-26	2	SN10-12, SN10-13

Table I. Relative *β-1,3-glucanase* expression in RT-PCR positive CG_1 plants .

Plant number	Average C_T (*β-1,3-glucanase*)	Average C_T (*β-tubulin*)	ΔC_T	$\Delta\Delta C_T$	$2^{-\Delta\Delta C_T}$
Non-transgenic	27.96±0.09	27.02±0.17	0.94±0.08	0±0.08	0.99 (0.94-1.05)
SN1-12	26.31±0.17	26.33±0.02	-0.02±0.15	-0.96±0.15	1.95 (1.75-2.15)
SN1-19	30.66±0.12	31.69±0.35	-1.03±0.23	-1.97±0.23	3.96 (3.34-4.59)
SN6-2	26.78±0.26	26.65±0.06	0.13±0.2	-0.81±0.2	1.76 (1.52-2.01)
SN6-5	30.09±0.15	27.91±0.20	2.18±0.05	1.24±0.08	0.42 (0.40-0.44)
SN10-12	27.37±0.15	28.53±0.26	-1.16±0.11	-2.1±0.11	4.14 (3.97-4.31)
SN10-13	29.10±0.07	30.31±0.15	-1.21±0.08	-2.15±0.08	4.44 (4.19-4.69)

Table J. Comparison of relative *β-1,3-glucanase* expression in leaves and roots of CG_1 transgenic plants before and after inoculation with Cf 09 pathotype.

Plant number	Average C_T (*β-1,3-glucanase*)		Average C_T (*β-tubulin*)		ΔC_T		$\Delta\Delta C_T$		$2^{-\Delta\Delta C_T}$	
	Before inoculation	After inoculation	Before inoculation	After inoculation	Before inoculation	After inoculation	Before inoculation	After inoculation	Before inoculation	After inoculation
**In leaves**										
Non-transgenic	27.40±0.92	25.42±0.18	27.49±0.34	25.39±0.25	-0.09±0.58	0.03±0.07	0±0.58	0±0.92	1.07 (0.66-1.49)	0.99 (0.95-1.04)
SN1-12	29.57±0.40	26.55±0.14	30.67±0.20	27.77±0.20	-1.1±0.20	-1.22±0.06	-1.01±0.20	-1.25±0.06	2.01 (1.73-2.29)	2.37 (2.28-2.47)
SN1-19	27.86±0.17	28.01±0.24	29.87±0.13	29.95±0.22	-2.01±0.04	-1.94±0.02	-1.92±0.04	-1.97±0.02	3.78 (3.68-3.89)	3.91 (3.69-4.13)
SN6-2	29.29±0.32	28.37±0.10	30.05±0.10	29.22±0.32	-0.76±0.22	-0.85±0.22	-0.67±0.22	-0.88±0.22	1.60 (1.36-1.85)	1.86 (1.58-2.14)
SN6-5	28.42±0.22	30.75±0.18	27.16±0.11	27.95±0.21	1.26±0.11	2.80±0.03	1.35±0.11	2.83±0.03	0.39 (0.36-0.42)	0.14 (0.13-0.14)
SN10-12	27.25±0.09	26.07±0.01	29.35±0.06	29.27±0.02	-2.1±0.03	-3.20±0.011	-2.01±0.03	-3.23±0.011	4.03 (3.94-4.12)	9.42 (9.35-9.49)
SN10-13	27.25±0.10	26.07±0.14	29.43±0.30	28.93±0.22	-2.18±0.07	-2.86±0.08	-2.09±0.07	-2.89±0.08	4.24 (4.04-4.44)	7.42 (7.01-7.42)
**In roots**										
Non-transgenic	26.90±0.15	26.02±0.12	26.83±0.20	25.42±0.15	0.07±0.05	0.60±0.03	0±0.05	0±0.03	0.99 (0.96-1.03)	0.99 (0.97-1.02)
SN1-12	24.79±0.16	26.04±0.09	24.82±0.11	25.79±0.03	-0.03±0.05	0.25±0.06	-0.10±0.05	-0.35±0.06	1.07 (1.03-1.10)	1.27 (1.22-1.32)
SN1-19	26.82±0.13	25.04±0.05	27.42±0.12	25.14±0.10	-0.60±0.01	-0.10±0.05	-0.67±0.01	-0.70±0.05	1.59 (1.58-1.60)	1.60 (1.56-1.64)
SN6-2	26.21±0.20	28.56±0.20	26.04±0.15	28.67±0.05	0.17±0.05	-0.11±0.15	0.10±0.05	-0.71±0.05	0.93 (0.90-0.96)	1.64 (1.47-1.81)
SN6-5	26.02±0.12	29.95±0.26	25.52±0.11	31.03±0.14	0.50±0.01	-1.08±0.12	0.43±0.01	0.48±0.12	0.73 (0.73-0.74)	0.71 (0.65-0.77)
SN10-12	23.80±0.30	25.09±0.07	24.48±0.10	27.50±0.01	-0.62±0.20	-2.41±0.06	-0.69±0.20	-3.01±0.06	1.61 (1.40-1.82)	8.04 (7.73-8.40)
SN10-13	23.80±0.13	25.09±0.05	24.60±0.12	27.06±0.10	-0.80±0.01	-1.97±0.05	-0.87±0.01	-2.57±0.05	1.82 (1.80-1.85)	5.94 (5.73-6.14)

Table K. Clonal propagation of red rot resistant CG_1 transgenic plants for raising CG_2, and PCR analysis of CG_2 plants.

CG_1 transgenic plant number	Number of billets planted	Number of billets sprouted	Designation of CG_2 plants	Number of PCR positive CG_2 plants	Designation of PCR positive plants
SN 10-12	17	7	C1-1<br>C1-2<br>C1-3<br>C1-4<br>C1-5<br>C1-6<br>C1-7	7	C1-1<br>C1-2<br>C1-3<br>C1-4<br>C1-5<br>C1-6<br>C1-7
SN 10-13	20	8	C2-1<br>C2-2<br>C2-3<br>C2-4<br>C2-5<br>C2-6<br>C2-7<br>C2-8	8	C2-1<br>C2-2<br>C2-3<br>C2-4<br>C2-5<br>C2-6<br>C2-7<br>C2-8

Table L. Relative *β-1,3-glucanase* expression in CG_2 plants.

Clone number	Average C_T (*β-1,3-glucanase*)	Average C_T (*β-tubulin*)	ΔC_T	$\Delta\Delta C_T$	$2^{-\Delta\Delta C_T}$
Non-transgenic	27.90±0.09	26.83±0.17	1.07±0.08	0±0.08	0.99 (0.94-1.05)
C1-1	25.82±0.17	26.02±0.02	-0.20±0.15	-1.27±0.15	2.42 (2.07-2.77)
C1-2	29.60±0.12	29.79±0.35	-0.19±0.23	-1.26±0.23	2.42 (2.04-2.80)
C1-3	24.52±0.26	24.82±0.06	-0.3±0.2	-1.37±0.2	2.60 (2.25-2.96)
C1-4	26.92±0.15	27.42±0.20	-0.5±0.05	-1.57±0.05	2.97 (2.86-3.07)
C1-5	26.04±0.15	26.52±0.26	-0.48±0.11	-1.55±0.11	2.93 (2.71-3.16)
C1-6	24.25±0.07	24.54±0.15	-0.29±0.08	-1.36±0.08	2.56 (2.42-2.71)
C1-7	24.60±0.07	24.80±0.15	-0.2±0.08	-1.27±0.08	2.41 (2.28-2.54)
Non-transgenic	26.66±0.17	27.16±0.09	-0.50±0.08	0±0.08	0.99 (0.94-1.05)
C2-1	26.06±0.15	27.80±0.07	-1.74±0.08	-1.24±0.08	2.36 (2.23-2.49)
C2-2	26.15±0.20	27.84±0.15	-1.69±0.05	-1.19±0.05	2.28 (2.20-2.36)
C2-3	26.14±0.26	27.94±0.15	-1.80±0.11	-1.30±0.11	2.47 (2.28-2.65)
C2-4	26.02±0.35	27.81±0.12	-1.79±0.23	-1.29±0.23	2.47 (2.08-2.86)
C2-5	26.08±0.15	27.87±0.07	-1.79±0.08	-1.29±0.08	2.46 (2.31-2.58)
C2-6	26.01±0.06	27.90±0.26	-1.89±0.02	-1.39±0.02	2.62 (2.38-2.86)
C2-7	26.17±0.20	27.96±0.15	-1.79±0.05	-1.29±0.05	2.44 (2.36-2.53)
C2-8	26.06±0.15	27.96±0.07	-1.90±0.08	-1.40±0.08	2.63 (2.49-2.77)

Table M. Identification of conserved active site residues in the catalytic domain of the protein

**4PEY**	**3EQN**	**AIC32930**
GLN174	GLN176	GLN188
TRP446	TRP572	TRP566
ASP449	ASP575	ASP569
GLU480	GLU610	GLU628
HIS481	HIS611	HIS606
TRY505	TRP636	TRY631
